# Impact sculpting of the early martian atmosphere

**DOI:** 10.1126/sciadv.adm9921

**Published:** 2024-09-11

**Authors:** Oliver Shorttle, Homa Saeidfirozeh, Paul Brandon Rimmer, Vojtĕch Laitl, Petr Kubelík, Lukáš Petera, Martin Ferus

**Affiliations:** ^1^ Institute of Astronomy, University of Cambridge, Madingley Road, Cambridge CB3 0HA, UK.; ^2^ Department of Earth Sciences, University of Cambridge, Downing Street, Cambridge CB2 3EQ, UK.; ^3^ J. Heyrovský Institute of Physical Chemistry, Czech Academy of Sciences, Dolejškova 3, CZ 18223 Prague 8, Czech Republic.; ^4^ Cavendish Laboratory, University of Cambridge, JJ Thompson Avenue, Cambridge CB3 0HE, UK.; ^5^ Faculty of Science, University of Antwerp, Groenenborgerlaan 171, BE-2020 Antwerpen, Belgium.; ^6^ Department of Inorganic Chemistry, Faculty of Science, Charles University, Hlavova 8, Prague, Czech Republic.

## Abstract

Intense bombardment of solar system planets in the immediate aftermath of protoplanetary disk dissipation has played a key role in their atmospheric evolution. During this epoch, energetic collisions will have removed substantial masses of gas from rocky planet atmospheres. Noble gases are powerful tracers of this early atmospheric history, xenon in particular, which on Mars and Earth shows significant depletions and isotopic fractionations relative to the lighter noble gasses. To evaluate the effect of impacts on the loss and fractionation of xenon, we measure its ionization and recombination efficiency by laser shock and apply these constraints to model impact-driven atmospheric escape on Mars. We demonstrate that impact bombardment within the first 200 to 300 million years of solar system history generates the observed Xe depletion and isotope fractionation of the modern martian atmosphere. This process may also explain the Xe depletion recorded in Earth’s deep mantle and provides a latest date for the timing of giant planet instability.

## Teaser

The early atmosphere of Mars records the solar system’s early and intense bombardment period.

## INTRODUCTION

Noble gases are a key tracer of the early loss and gain of planetary atmospheres. Their inert nature makes their abundances and isotopic composition ideal archives of fractionation processes that occur during volatile loss and redistribution in planets. Xenon, in particular, has proven an enigmatic carrier of information on atmospheric evolution, showing anomalous depletions and mass fractionations in the atmospheres of both Earth and Mars (*1*). On Earth, this has led to the identification of the “missing Xe” problem, where Xe is noted to be unusually depleted in Earth’s atmosphere given the element’s high mass (*2*). Associated with this depletion is a large mass-dependent fractionation of Xe isotopes, greater than that seen among the isotopes of Kr despite their being lighter, and is a signal that has been constrained to have grown to its modern value over the first 2 billion years (Gyr) of Earth history (*3*–*5*).

**Fig. 1.**
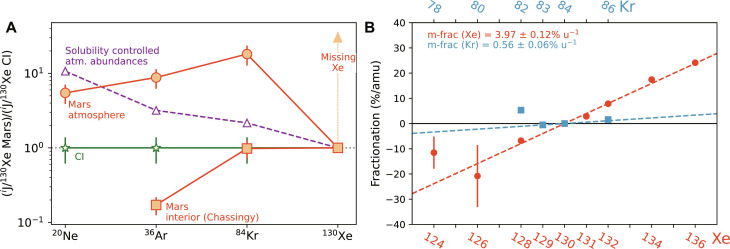
Key characteristics of the martian noble gas reservoirs. (**A**) Elemental abundances of noble gases in the martian interior [as represented by Chassigny (*75*)] and atmosphere (*1*, *76*), ratioed to the abundance of Xe, relative to the elemental ratio in CI chondrites (*75*). Xe is noticeably depleted compared to the other noble gases. The relative noble gas abundances that would be produced in an atmosphere formed from a two-stage degassing process are shown as triangles. First, during the magma ocean stage, noble gases are distributed between the atmosphere and interior according to their relative solubilities (*60*), after which the initial atmosphere is lost. Second, subsequent volcanic degassing quantitatively removes noble gases from the melted sources region as magmas degas at low pressure. In this way, the most soluble gasses are dominant in the atmosphere that is restored. (**B**) The isotopic fractionations of Xe (red circles) and Kr (blue squares) in Mars’ atmosphere [data from (*6*)]. Fractionations were calculated per atomic mass unit, considering only the heaviest four isotope ratios of Xe (131/130, 132/130, 134/130, and 136/130) and the 83/84 and 84/84 ratios of Kr. All reported errors are 1 sigma.

Like Earth, Mars’ atmosphere evidences preferential Xe loss and isotopic fractionation compared with other noble gases [e.g., (*6*) and Fig. 1]. The isotopic fractionation of Xe is of similar magnitude between the two atmospheres (*7*); however, the atmospheres began their evolution from different starting compositions, solar Xe in the case of Mars (*8*) compared with U-Xe for Earth [itself a mixture of cometary and chondritic Xe (*1*, *9*)]. The isotopic fractionation of Xe in the martian atmosphere must have occurred early: Data from two martian meteorites, ALH 84001 and NWA 11220, which sample Mars’ atmosphere at ∼4.2 and ∼4.4 Ga, respectively, indicate that fractionation to the modern value occurred rapidly in the first few hundred million years of the planet’s history (*10*, *11*).

An explanation for the progressive multibillion year isotopic fractionation of Earth’s atmospheric Xe has been provided by Zahnle *et al.* (*12*), who suggest that Xe escaped Earth in a photo-ionized hydrogen wind. In this model, Xe loss and fractionation occur sporadically over the first several billion year’s of Earth history and only terminate when atmospheric oxygenation sharply decreases the H_2_ mixing ratio of the atmosphere. Key to this model is the low ionization potential of Xe, the lowest among the noble gases, which means it is preferentially removed despite its high mass: Ionized Xe couples to open magnetic field lines and is dragged to space by ionized hydrogen. A similar model of Xe escaping in an ionized wind has recently been advanced to explain Xe’s loss from Mars’ atmosphere (*11*). In the case of Mars, observations require that the fractionation terminates early [before 4.2 Ga; (*10*)], which is suggested to occur because of either waning extreme solar ultraviolet (UV), diminished H_2_ sources on the planet, or cessation of the martian magnetic field (*11*). The latter scenario is disfavored because of evidence of magnetism in rocks younger than 4.2 Ga (*13*). Diminishing H_2_ sources on the planet may be inconsistent with requirements for Mars’ transiently wet early climate, which recent modeling has suggested needed episodic reducing conditions (i.e., high H_2_) for over a billion years into the planet’s life (*14*). This is long after fractionation of Xe in the atmosphere is observed to have ceased (*10*).

Intriguingly, recent work has indicated that a separate epoch of preferential Xe loss must have also occurred much earlier in Earth’s history, earlier than the slow loss recorded in its atmosphere over ∼2 Gyr (*15*). Earth’s deep mantle may, therefore, have captured evidence of a separate Xe (and wider volatile) loss episode within its first few hundred million years (Myr) (*16*), an episode contemporaneous with Mars’ observed Xe loss (*10*). Together, these observations suggest that additional processes(s) may have operated across the solar system early in the life of rocky planets to drive the rapid loss of volatile elements. As Xe records preferential depletion by these processes, models other than the classic hydrodynamic escape scenarios are required [e.g., (*17*)], as these would favor the preferential loss of light gases over Xe.

Although long considered in the context of atmospheric loss (*18*), impact bombardment is one mechanism for isotopic fractionation of atmospheres that has received little attention. The impact bombardment of planets is widely recognized as an important process shaping their formation and evolution (*19*). During planetary growth, accretionary impacts are the primary mechanism by which rocky planets gain mass (*20*–*22*), with the most marked and recent known example in the solar system being the collision of a Mars-sized impactor with the proto-Earth to form the Moon (*23*). One peak in impact rates onto planets likely occurs following the dissipation of their natal protoplanetary disk, which in the solar system occurred at ∼3 Myr after its birth (*24*). Later peaks in impact fluxes would coincide with dynamical reorganisations of the solar system giant planets (*25*).

The abundance and rate of impacts in this early epoch have been constrained by cratering records on the Moon (*26*), Mars (*26*–*28*), and by the highly siderophile element (HSE) abundances of the terrestrial and martian mantles (*29*). However, the timing of any increased bombardment following giant planet instability remains uncertain: Initially linked to clustered ages of lunar rocks between ∼3.5 and −4.2 Ga (*30*, *31*), it now seems likely that the lunar observations at least can be explained with a gradual tailing-off of accretion (*32*) and that any instability occurred earlier (*33*–*35*). Impact bombardment could significantly affect atmospheric retention on rocky planets (*36*–*38*), being capable of both delivering and removing volatile elements depending on impactor size. In this case, the atmospheric evolution of the terrestrial planets may itself be a record of this early bombardment history.

**Fig. 2.**
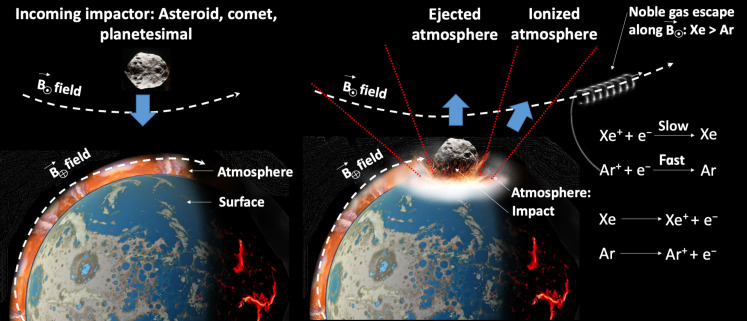
Model framework for describing Xe loss and fractionation from the martian atmosphere. Impacts eject all material in a central cone, with an annulus of partially ionized material escaping the atmosphere according to whether ions survive to high altitude to connect with Mars’ open magnetic field lines (*77*).

Impacts have been presumed to not affect the fractionation of Xe in planetary atmospheres because of their propensity to remove atmosphere from their target indiscriminately (*18*, *37*, *39*). However, while a hypervelocity impactor is moving through the atmosphere, it produces a high temperature ionized shock (a quasi-neutral plasma over 10^4^ K) in a wide region around it (*40*). This plasma ends up thrown away from the planet’s surface to high altitude [Fig. 2 and (*37*)]. While for a sufficiently large impactor some atmosphere in this cone-shaped region will be directly ejected, the planet will also retain a proportion of the ionized atmosphere. The transiently ionized atmosphere from hypervelocity impacts, therefore, represents a potential mechanism of creating ionized Xe at heights in the atmosphere where it may couple to magnetic field lines and escape the planet. This is a scenario similar to that envisaged in (*12*) but with impacts rather than photons as both the source of the ionization and as the mechanism to move Xe up to high altitude (i.e., induce enhanced vertical mixing). Xe loss by impacts would decouple the history of Xe from the H_2_ mixing ratio of a planetary atmosphere, whereas Cassata *et al.* and Zahnle *et al.* (*11*, *12*) have emphasized the importance of hydrogen for ionizing the Xe and transporting it vertically. Impacts achieve both of these processes, ionizing Xe through high temperature plasma and ballistically lofting it in the atmosphere, without recourse to specific background atmospheric H_2_ mixing ratios. This is perhaps useful in the context of the requirements of Mars’ early climate (*14*) and would present a new dependence for the timescale of Xe’s evolution, occurring during epochs of intense impact bombardment. Such an association between impacts and Xe isotopic evolution has been noticed for Earth [figure 7c of (*4*)], although our present work does not seek to explain Earth’s more protracted Xe-loss history.

For such a scenario to explain the relative Xe abundance and isotope composition of Mars’ atmosphere, it is key that Xe, compared with the other noble gases, be preferentially affected by impact-driven ionization and escape. Two conditions would contribute to preferential Xe loss and isotope fractionation: first, if the high temperatures of an impact plasma ionized Xe appreciably more than other noble gases; second, preferential Xe loss would be aided by the recombination rate of Xe being lower than that of the other noble gases, given that the ionized Xe will also need to be transported from the site of the shock to a height where it can couple to open magnetic field lines and be lost. In particular, for this second condition, we are interested in the rate of the reaction

$$Xe^{+}+e^{-}+M\to \text{Xe}+M$$ (1)

where M is any neutral third body, versus the rate of equivalent reactions for other noble gases. If one or both of these conditions were met without the other actively disfavoring Xe ionization, then more Xe remains ionized once lofted up by impact than other noble gases, aiding Xe escape.

Experiment and theory constrain the ionization energy for xenon to be lower than for any other noble gas (*41*) and that the rate at which ionized xenon recombines with its electron is slower than for the other noble gases (*42*). However, the experiments that give these rates either measure the thermal ionization rate and calculate the rate of the reverse reaction (*43*, *44*) or have only measured the forward electron-ion recombination rates (*45*, *46*). A final limitation of the current data is that no ion-electron recombination experiments have been performed for noble gases in the context of a planetary atmosphere; the role of geologically relevant background gasses in Xe recombination is therefore poorly constrained.

To provide new constraints on the key parameters of impact-driven ionization, we performed laser shock experiments to simulate impactor entry into planetary atmospheres. These give us direct experimental constraints on the thermal ionization of noble gases during an impact and the recombination rates for those ions during, and immediately after, the impact. We obtain new estimates of ionization fractions and recombination rates for Xe, using Ar as a reference noble gas with a higher ionization threshold. Combining the Xe ionization parameters with a simple model of impact-driven atmospheric loss [based on (*37*)], we then explore the viability of impacts as the exclusive mechanism explaining Mars’ history of Xe isotopic fractionation. We find that impacts can explain the early onset and magnitude of Xe isotopic fractionation in the martian atmosphere, subject to the initial mass of the atmosphere and the specific bombardment history Mars experiences. While other loss processes were likely simultaneously operating on planets to drive atmospheric loss and Xe fractionation (*11*), our results suggest that impact sculpting of the early martian atmosphere was likely important. In this context, the recently discovered Xe depletions in the deep Earth (*15*) indicate that impact-driven loss processes may have operated early in its history as well.

## RESULTS

### Laser shock experiments

**Fig. 3.**
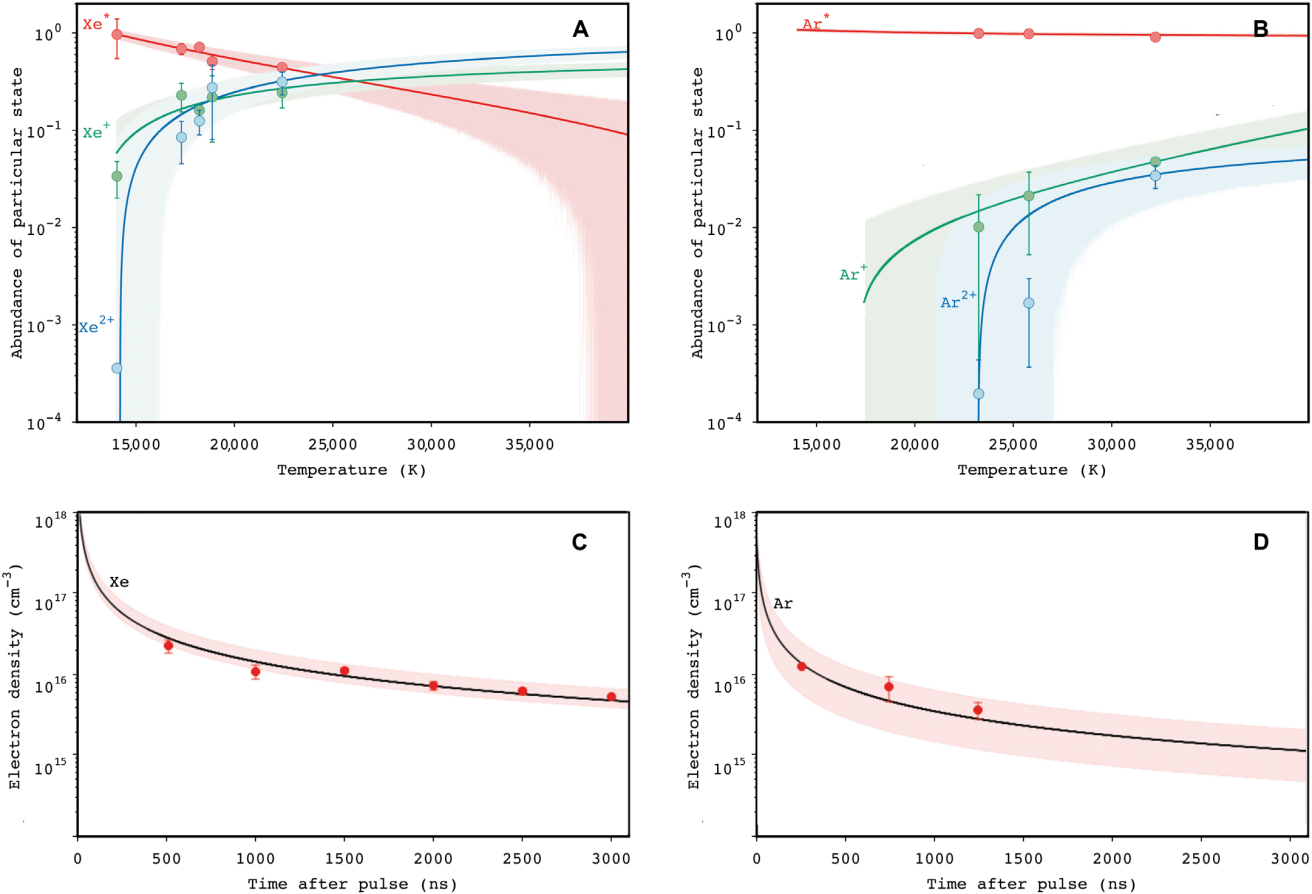
Experimental results showing the relative ionization efficiency and recombination rates of Xe and Ar during laser shock. (**A** and **B**) Abundance of particular Xe/Ar states as a function of temperature for experiments run with pure Xe and Ar at a total pressure of ∼0.17 bar at 1500 ns following the laser pulse. Superscript star indicates neutral species, and superscript plus indicates charged or multiply charged species. The solid lines show the fit from simulations of the excited states, with shaded envelopes giving the 97.5% confidence interval from data uncertainty: Simulations were based on modeling Saha ionization equilibrium with varying temperature (see Materials and Methods for details). (**C** and **D**) Electron density variation with time for pure Xe/Ar mixtures following the initial laser pulse. Points were taken from experimental data, and solid curves show fits of Eq. 2 to the data to estimate *k*_r_, the recombination rate (full details in Materials and Methods).

Laser shock experiments were conducted as described in (*47*) to estimate key parameters of atmospheric ionization during hypervelocity impact. Mixtures of Xe-H_2_ and Ar-H_2_ were heated by short pulses of high-energy laser to create a plasma. Ar, as a noble gas with higher ionization threshold than Xe (*12*), was chosen so that the relative ionization efficiencies and recombination rates between the two gases could be compared. Spectra were recorded from ∼200 ns after the initial laser-induced plasma was formed, and from these, the Xe and Ar ionization fractions were estimated (see Materials and Methods for the complete description; Fig. 3).

The laser shock experiments show that Xe is more strongly ionized than Ar (Fig. 3, A versus B): For a plasma temperature of 2 ×10^4^ K, well within the range of temperatures atmosphere is expected to be heated to during impact (*40*), ∼40% of the Xe is present as either singly or doubly charged ions (Fig. 3A); this compares with less than 1% of Ar being present as charged ions at the same temperature (Fig. 3B). With increasing temperature, more Xe and Ar are ionized, but at all temperatures investigated, Xe remains over an order of magnitude more ionized than Ar.

The second important result from the experiments is an estimate of the relative recombination rates of Xe and Ar. Recombination rates were calculated by fitting the following equation to the data

$$n_{e}\left(t\right)=\frac{n_{e}\left(0\right)}{1+n_{e}\left(0\right)k_{r}t}$$ (2)

where *n*_e_(*t*) (cm^−3^) is the electron density at time *t* (s), *n*_e_(0) is the electron density immediately following the laser pulse (at which time the gas is assumed to be fully ionized), and *k*_r_ (cm^3^ s^−1^) is the recombination rate. The fits of Eq. 2 to these time series are shown in Fig. 3 (C and D). A recombination rate of 6 ± 2.5 × 10^−11^ cm^3^ s^−1^ is found for Xe and a higher rate, 4 ± 1 × 10^−10^ cm^3^ s^−1^, for Ar. Xe recombination is, therefore, found to occur significantly more slowly than Ar recombination.

The results shown in Fig. 3 (A and B) are for ionization of pure Xe or Ar gases. However, experiments were also conducted for various mixtures of Xe and Ar with H_2_ gas. Each of these species has different ionization energies, *I* (eV). Since H_2_ will have been much more abundant than Xe in Mars’ early atmosphere (*14*, *48*) and that charge exchange between the ionization products of H_2_ (*I* = 15.4 eV) and Xe (I = 12.1 eV) has been proposed as a mechanism for ionizing Xe (*12*), we look into the dependency of Xe and Ar (*I* = 15.8 eV) thermal ionization yield and recombination rates on H_2_ concentrations. The results of these experiments (figs. S2 and S3) show that increasing the H_2_ mixing ratio has only a small effect on the electron density and the recombination rate of Xe and Ar. This provides reassurance that in an early planetary atmosphere, whether it has a high mean molecular weight, e.g., is CO_2_ ± H_2_O ± N_2_ dominated, or is H_2_-rich, Xe’s ionization behavior will be well described by the results for the pure gasses presented in Fig. 3.

Overall, the laser shock experiments indicate that atmospheric plasma generated by hypervelocity impact will contain more ionized Xe than Ar and that the Xe will remain ionized for longer than the Ar. The ionization behavior of Xe during impact compared to that of Ar therefore favor Xe’s transport to high altitude while still ionized and hence its potential loss from the planet along magnetic field lines. We next take the parameters derived from these experiments and use them in a simple model of impact-driven Xe and Ar escape to investigate whether the observed isotopic fractionation of Xe can be generated and on what timescale (Fig. 1).

### Modeling impact-driven Xe loss and fractionation

**Fig. 4.**
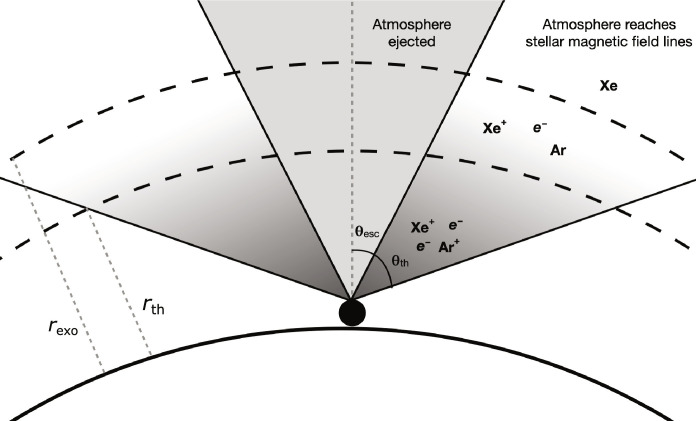
Diagram of xenon impact ejection and escape. Cross sections are shown for the two cones of ejected (center) and ionized and conditionally lost atmosphere (either side) for our model. Atmosphere in the solid gray cone subtended by θ_esc_ is ejected from the atmosphere by the impact, is unbound by gravity, and moved into the exosphere >*r*_exo_ (km). Atmosphere in the gradient gray cone subtended by θ_th_ passes the homopause and reaches the thermosphere *r*_th_ (km). Species that remain ionized at this point (e.g., Xe and Ar) will be confined by the stellar magnetic field and escape the atmosphere.

A schematic of how we model impact-driven loss of Xe to occur is presented in Fig. 4. This treatment of impact-driven loss closely follows that developed in (*37*), in which the atmosphere is ejected by planetesimal impact above a tangent plane with respect to the planet’s surface. An atmospheric loss event involves an impactor of a given mass entering the atmosphere with some velocity. The impactor ejects all atmosphere, i.e., all gases equally, in a solid cone around its entry axis. A second wider cone extends around this central volume of the lost atmosphere; this outer cone is ionized and ejected upward but without sufficient velocity that it will be ballistically ejected from the planet’s gravitational well. Instead, we model gas in this cone of material as being conditionally lost subject to the ionization state of the gases at the point they reach the homopause.

Before proceeding, we emphasize two important simplifications, or assumptions, of our model’s approach. First, we assume that the overall atmospheric pressure during impact bombardment is constant. In the context of explaining Xe loss and fractionation, this would correspond to a scenario in which impact processing of the martian surface releases major atmospheric constituents such as CO_2_ and H_2_O, by melting rock or destabilizing ices, equivalent to the mass of atmosphere lost. Second, we assume that the impactors deliver no noble gases. This second assumption would correspond to either the impactors themselves containing negligible noble gases when compared to the amount removed (e.g., they may be differentiated and degassed) or that the gases they do contain are volatilized and ejected during impact in the escaping cone (Fig. 4) along with the target atmosphere. We revisit both of these assumptions in Discussion.

As we saw in the previous section, Xe is ionized more completely and recombines more slowly than Ar. The key question for impact-driven loss and mass-dependent fractionation of Xe is then to quantify how much more efficiently Xe will be lost compared with the other noble gases (i.e., to explain the observations in Fig. 1A) and how much more efficiently will the lighter isotopes of Xe be lost than heavier isotopes (i.e., to explain the observations in Fig. 1B). In the framework of impact-driven escape, these efficiencies are set by the relative degrees of ionization of the noble gases at the point the impact plasma reaches the homopause. To calculate this, our simplified model defines a single pressure in the atmosphere at which the plasma is made. From this point, it calculates the mass of gas in the outer cone that will be ejected upward but only conditionally lost and how long it takes that gas to reach the homopause. Knowing the ionization yields (Fig. 3, A and B) from impact and the recombination rates (Fig. 3, C and D), Eq. 1 can be applied to convert ions back to neutral species during the time it takes to move the impact-induced plasma from its site of formation to the homopause.

The time to the homopause, therefore, emerges as key in setting the efficiency with which impacts can drive Xe (and Ar) loss. We assume that the ionized xenon and argon travel a distance *h* with a time of flight inversely proportional to the square root of their mean molecular weights. Ions, therefore, are mass segregated in the ionized gas during their time of flight and have more time to become neutralized and then settle back into the lower atmosphere, the heavier they are. We set the time of the flight to

$$t_{\text{fl}}=\sqrt{\frac{h}{2g}}\;\sqrt{\frac{\mu \left(X\right)}{\mu_{a}}}$$ (3)

where *g* is the gravitational acceleration, μ(X) is the mean molecular weight of species X, and μ_a_ is the mean molecular weight of the atmosphere. As xenon is heavier than argon, Eq. 3 favors Xe retention and Ar loss. However, as we have already seen, the recombination rates and initial ionization fractions counteract this effect. The differential time of flight does, though, mean that the heavier isotopes of Xe may be separated from the lighter isotopes and preferentially retained (Fig. 1B).

Once the partially ionized gas has reached the homopause, we assume that all species remaining ionized are lost. For the purposes of these calculations, we are only tracking ionization-dependent loss of Xe and Ar, but we also track the total atmospheric mass lost by direct ejection (central cone shown in Fig. 4).

Our model considers the cone of atmosphere ejected past the homopause into the thermosphere, subtracting out the cone of atmosphere that is entirely ejected (Fig. 4). The fraction of the non-ejected atmosphere that remains Xe^+^ is removed from the atmosphere. This mass loss can be expressed as the depleted mass of Xenon [$M_{D}$(*Xe*), kg]

$$M_{D}\left(\mathrm{Xe}\right)=\frac{M_{0}\left(\text{Xe}\right)f_{0}\left(\text{Xe}\right)M_{T}\left(\text{Xe}\right)}{M_{0}\left(\text{Xe}\right)f_{0}\left(\text{Xe}\right)+M_{T}\left(\text{Xe}\right)}$$ (4)

where *f*_0_(Xe) is the initial mixing ratio of Xe below the homopause, $M_{T}$ (Xe) (kg) is the total mass of Xe below the homopause, and

$$M_{0}\left(\text{Xe}\right)=\frac{2N_{0}}{\beta -2}\;\left(\frac{\mu \left(\text{Xe}\right)}{\mu_{a}}\right)\chi_{0}\left(Xe^{+}\right)e^{-\Lambda_{r}}\;\rho_{\text{imp}}R_{0}^{3}$$ (5)

χ_0_(*Xe*^+^) is the initial fraction of Xe that is ionized, μ_a_ is the mean molecular weight of the atmosphere, μ(Xe) is the mean molecular weight of Xenon, ρ_imp_ (g cm^−3^) is the mass density of the impactor, and *N*_0_ and *R*_0_ (km) are the number and size of impactors found by integrating the impactor size distribution

$$\mathrm{dN}=\beta N_{0}{\left(\frac{R_{0}}{r_{\text{imp}}}\right)}^{\beta }\;\frac{dr_{\text{imp}}}{r_{\text{imp}}}$$ (6)

where *r*_imp_ (km) is the size of a given impactor and β > 2 is an integer that sets the power law of the size distribution.

The quantities *R*_0_ and Λ_r_ are parameterized in terms of the free and experimentally constrained parameters of the height of the homopause (*h*, km), the radius of the planet (*R*_p_, expressed in Earth radii, *R*_⊕_), surface gravity (*g*, m s^−2^), surface pressure (*p*, bar), surface temperature (*T*, K), ionization fraction (*f*_e_), and recombination rate (*k_r_*, cm^3^ s^−1^), as so

$$\begin{array}{c} R_{0}\left(\text{km}\right)=28.45{\left(\mu_{a}\right)}^{1/3}{\left(\frac{1\text{gc}m^{-3}}{\rho_{\text{imp}}}\right)}^{1/3}\\ {\left(\frac{h}{1\text{km}}\right)}^{1/6}{\left(\frac{R_{⊕}}{R_{p}}\right)}^{1/6}\left(\frac{1ms^{-2}}{g}\right){\left(\frac{p}{1\text{bar}}\right)}^{1/3}{\left(\frac{T}{300\;K}\right)}^{2/3} \end{array}$$ (7)

$$\begin{array}{c} \Lambda_{r}=\left(5.4\times 10^{20}\;cm^{-3}s\right)\;f_{e}k_{r}\left(\frac{p}{1\text{bar}}\right)\\ \left(\frac{300K}{T}\right){\left(\frac{h}{1\text{km}}\right)}^{1/2}{\left(\frac{1ms^{-2}}{g}\right)}^{1/2}{\left(\frac{\mu \left(\mathrm{Xe}\right)}{\mu_{a}}\right)}^{1/2} \end{array}$$ (8)

Details of this model are given in the Supplementary Materials.

**Fig. 5.**
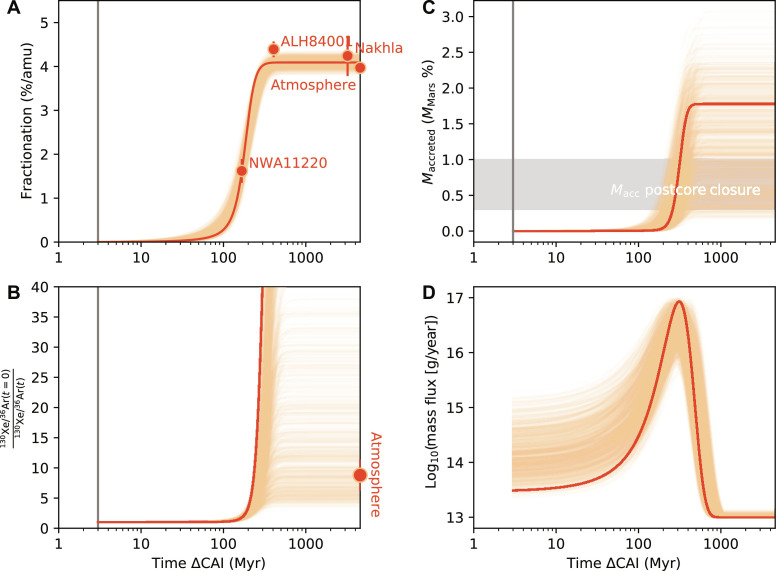
The evolution of martian Xe during impact bombardment. (**A**) The isotopic fractionation of Xe through time in the model, compared to modern and ancient estimates of the atmospheric Xe isotope fractionation (*6*, *10*, *11*, *78*). (**B**) The evolution of the Xe/Ar ratio during impact-driven loss. Impact bombardment in the best fitting model leaves the Xe/Ar ratio below modern values, allowing for subsequent solar wind–driven loss and fractionation of Ar (*53*) to drive the atmosphere to modern values. (**C**) The cumulative mass delivered to Mars in impactors, compared to the estimate of accreted mass postcore closure from HSEs (*61*, *62*). (**D**) The impact rate histories of solutions consistent with the Xe mass fractionation data. In all cases, the best fitting model (maximum likelihood estimate) is shown in a dark red solid line.

The forward model described above is combined with a Bayesian Monte Carlo inversion routine to estimate the values for the parameters of interest (see Table 1 for a complete list of parameters and the priors they were assigned in the modeling) by fitting the model to the Xe mass fractionation record [Fig. 5A and (*11*)]. Bayesian inversion is performed using MultiNest, a Monte Carlo nested sampling algorithm (*49*, *50*) via pyMultiNest (*51*). The model includes eight parameters, four of which are included as nuisance parameters to be marginalized over for error propagation. Four parameters are of key geological interest as they speak to the solar system and martian history, and these are discussed in more detail below. The value these parameters take when matching the observations is, therefore, a key test of the model’s validity; they offer potential for testing against independent geological records.

**Table 1.** Parameters estimated in the Bayesian inversion, including their prior values and distributions. Priors are prescribed as uniform (“U”) or log uniform (“LU”).

Parameter	Units	Description	Prior
Parameters of geological interest			
*p*_surf_	bar	Surface pressure	LU(10^−3^,10^1^)
*C*_peak_	–	Mass flux increase by 10^*C*_peak_^ above background	U(0,4)
*t*_peak_	Ga	Time of peak mass flux	U(4.564,4.000)
Δ*t*	Gyr	Time window of increased mass flux	U(0.15,0.25)
Parameters included for error propagation			
*h*	km	Height of escape	U(50,1000)
*p*	bar	Impact pressure in atmosphere	LU(10^−6^, *p*_surf_)
*T*	*K*	Temperature of atmosphere between impact and *h*	U(200,1000)
*f_e_*	–	Ionization fraction of background atmosphere	LU(10^−12^,10^0^)

The first of these parameters is atmospheric surface pressure, *p*_surf_ (bar). This defines the mass of the martian atmosphere, and therefore its starting Xe inventory (via the prescribed Xe mixing ratio). In the context of impact-driven escape, more Xe in the atmosphere initially will require more impacts to remove. The surface pressure of the martian atmosphere is also of direct relevance to the planet’s climate history. This leads to the second parameter, *C*_peak_, a factor modifying the exponent of the mass flux of impactors onto the planet. A highly simplified prescription for impactor flux is used, in which a constant background flux is perturbed by a single peak, of height 10^*C*_peak_^ above background (see Materials and Methods for full description). The timing of this peak in impactor flux is controlled by the third parameter, *t*_peak_, and its width by the fourth parameter Δ*t*. As we will see, in this simple model the time of the peak impact flux is a key parameter in fitting the temporal evolution of martian Xe loss and fractionation (*11*).

The net effect of the range of *t*_peak_ and Δ*t* permitted in the inversion (Table 1) is for there to be a declining impactor flux over the first ∼1 Gyr of solar system history. The integrated impactor flux gives the total mass gained by the planet during impact bombardment and can in principle be tested against cratering records and HSE-derived estimates of postcore-formation mass accretion.

The fit of the model to the time-resolved record of Xe isotopic fractionation data are shown in Fig. 5A. The red line records the solution calculated from the median of the parameters’ posterior distributions and provides a close fit to the ∼4 Gyr of Mars’ Xe mass fractionation. In particular, the model is able to reproduce the rapid in-growth of mass-fractionated Xe in Mars’ atmosphere in the first few hundred million years of its history.

The single modern constraint on the factor by which the atmospheric Xe/Ar ratio has been decreased below its initial value, $\left(\frac{{\;}^{130}\text{Xe}{/}^{36}\text{Ar}\left(t=0\right)}{{\;}^{130}\text{Xe}{/}^{36}\text{Ar}\left(t\right)}\right)$ , was not included in the model fit; however, solutions range from around the modern atmospheric value to significantly higher degrees of fractionation [Fig. 5B; median $\left(\frac{{\;}^{130}\text{Xe}{/}^{36}\text{Ar}\left(t=0\right)}{{\;}^{130}\text{Xe}{/}^{36}\text{Ar}\left(t=4.5\;\text{Gyr}\right)}\right)$ of ∼100]. This wide distribution on the level of decrease of the Xe/Ar ratio, yet tightly constrained Xe mass fractionation histories, comes from the sensitivity of the loss of Xe and Ar to their relative recombination rates; a factor that does not affect consideration of Xe isotopes alone, where a given recombination rate can be compensated for through other parameters. For the model’s consistency with the modern atmosphere, subsequent loss processes may remove atmospheric Ar over Mars’ history and thus lower an initially high $\left(\frac{{\;}^{130}\text{Xe}{/}^{36}\text{Ar}\left(t=0\right)}{{\;}^{130}\text{Xe}{/}^{36}\text{Ar}\left(t\right)}\right)$ down to the observed value (*52*). Given the median $\left(\frac{{\;}^{130}\text{Xe}{/}^{36}\text{Ar}\left(t=0\right)}{{\;}^{130}\text{Xe}{/}^{36}\text{Ar}\left(t=4.5\;\text{Gyr}\right)}\right)$ predicted in our model, a loss of ∼90% of the postimpact Ar inventory would evolve Mars’ atmosphere to its modern Xe/Ar ratio. This is consistent with independent models explaining the modern ^36^Ar/^38^Ar ratio of the martian atmosphere (a ratio affected at less than the part per million level by impact bombardment), which suggest up to 95% of Mars’ atmospheric argon inventory may have been removed gradually over its history (*53*).

## DISCUSSION

Our experimental and numerical results demonstrate that impact bombardment could have driven the preferential loss and isotopic fractionation of Xe in the atmosphere of Mars. Critically, this could only have occurred early in the lifetime of the solar system, while the mass flux of impactors onto planets was still substantial: the ability of the models to match the observed rapid in-growth of Xe isotope fractionation followed by stasis (Fig. 5C), directly emerges from this history. Specifically, the models require an early peak in impactor flux followed by rapid decline in impact rate over the first few hundred million years of solar system history (*54*).

Two important assumptions the model makes are of a constant atmospheric pressure during bombardment and no Xe delivery. Atmospheric bombardment is expected to erode atmospheres (*37*), inferred from the same theoretical approach we take to predict Xenon escape. This erosion can be counterbalanced by delivery of volatiles (*55*) or by degassing from the magma generated at the impact site. For simplicity, we assume that these different sources (delivery and impact-generated magma degassing) and sinks (impact erosion), when summed over all species lead to a stable, albeit low pressure, atmosphere, and that the sources provide negligible Xenon [the convergent state of an atmosphere under bombarded (*55*)]. Below, we consider the effect of relaxing each of these assumptions.

**Fig. 6.**
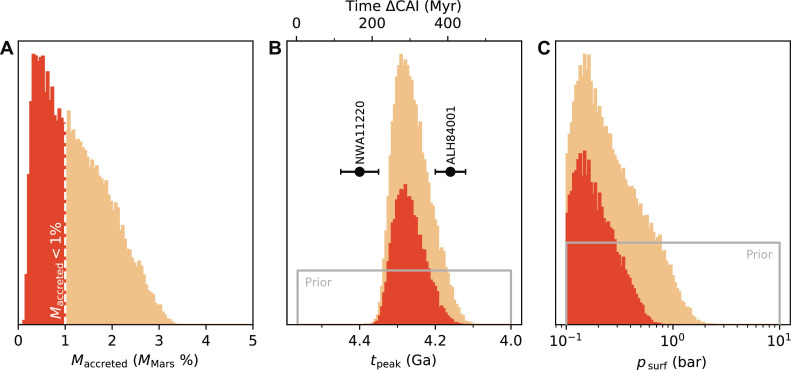
Posterior distributions of parameters consistent with HSE constraints on accreted mass. (**A**) The posterior distribution of accreted mass to Mars [*M*_accreted_ (*M*_Mars_%)], following impact bombardment sufficient to produce the observed Xe isotopic fractionation. Approximately 45% of solutions, given the choice of priors, fall at or below the ∼1% (*M*_Mars_) limit on postcore-closure mass addition inferred from HSEs (*61*, *62*), and these are shown in light orange. In dark red are the solutions where less than 1% the mass of Mars was accreted, and the observed Xe isotopic fractionation was still produced. (**B**) The posterior distribution of the onset of impact bombardment-driven isotopic fractionation. The gray line shows the uniform prior, and the histograms show the resultant prior (light orange) and subset of the posterior that matches the accreted mass constraint (dark red). (**C**) The posterior distribution of surface pressure during the impact bombardment that produces the observed Xe isotopic mass fractionation (light orange). Dark red identifies the results consistent with constraints on accreted mass.

Allowing changes in the atmospheric surface pressure can enhance impact-driven isotope fractionation. If the atmosphere is being eroded, then the Xenon depletion will be increased because, as we have shown (Fig. 6C), Xenon ionization and escape are more efficient when the surface atmospheric pressure is lower. If the atmosphere is growing, due to efficient delivery or efficient magma degassing, then our mechanism will be frustrated. Further work to constrain these sources and sinks and explore potentially observable implications of these constraints will be needed to inform whether and how this assumption should be relaxed. However, the evidence for a low pressure atmosphere on early Mars suggests significant atmospheric growth during this bombardment epoch was unlikely (see discussion below).

We consider the assumption of no Xe delivery during bombardment a reasonable starting point for modeling the following reasons.

1) Impactor Xe will be lost along with the cone of atmosphere it is interacting with. The efficiency of impactor Xe partitioning between loss and delivery is difficult to predict with our present state of knowledge, because it will depend on the dynamics of the impact event. If the delivered Xenon is included within the cone of matter that is ejected from the atmosphere (*37*), then it will necessarily not contribute to the atmospheric budget. A complete physical model would be required to calculate the partitioning of impactor Xe between loss and delivery, which is beyond the scope of this work, but qualitatively has the effect of diminishing the potential of Xe delivery compared with the impactor’s initial inventory;

2) For a surface atmospheric pressure of 0.1 bar, Xenon will only be added if the delivery brings more Xenon than 10^−11^ g/g [i.e., more Xe contained in the meteorite than is lost above the homopause; (*56*)]. If we consider the higher surface pressures permitted by the model (Fig. 6C), then even higher impactor Xe concentrations are required to perturb the atmospheric Xe budget. By comparison to a conservative 10^−11^ g/g value though, carbonaceous chondrites bracket this, having a range of Xenon concentrations between (0.01 − 2.5) × 10^−11^ g Xe/g total. If the more Xenon-rich carbonaceous chondrites represent the average impactor composition during the tail end of accretion, then Xenon will be slowly increased by impact delivery. Ordinary chondrites, in contrast, have less than 10^−11^ g/g Xenon on average (*57*) and so will not contribute enough Xenon to substantially change our results. E-chondrites have even less Xenon, on average 5 × 10^−13^ g/g (*58*). Evidence points to Mars’ late accretion being dominated by ordinary- and enstatite chondrite-like material (*59*), suggesting that Xe delivery would have been unimportant even before consideration of point (*1*) above; and

3) Mars’ atmosphere is constrained to have started with a solar-like Xe isotopic composition (*8*), from which subsequent atmospheric evolution occurred. This observation rules out late delivery as a prominent contributor to Mars’ atmospheric Xe, because this would have delivered nonsolar chondritic Xe—impacts must have acted primarily to sculpt Mars’ initial solar-Xe inventory rather than add to it.

In addition to impactor delivery, impact-generated magma degassing is unlikely to provide significant Xenon to the atmosphere: Xenon’s insolubility in magmas (*60*) makes it likely that the Xenon concentration in the Mars’ crust/interior will be much lower than the Xenon abundance in the atmosphere. Overall then, we consider it most likely that Mars’ Xe inventory during this bombardment epoch was dominated by loss and fractionation not delivery or interior outgassing.

An important aspect of impact bombardment as a means of preferentially driving Xe loss and fractionation is that it does not depend on background H_2_ in a planet’s atmosphere. This is in contrast to previous work for Earth and Mars (*11*, *12*), in which Xe ionization by charge exchange and transport through the atmosphere is linked to atmospheric hydrogen content: such that in the case of the Earth the cessation of Xe fractionation is hypothesized to be linked to the oxidation of the atmosphere (*12*). Our experimental results showed limited sensitivity of Xe ionization and recombination to the presence of background H_2_, and the impact mechanism directly provides ballistic transport of Xe through the atmosphere to the ionosphere. Hence, impact-driven fractionation and Xe loss do not make specific predictions, nor have specific requirements for, the evolution of the background atmospheric composition. A corollary of this is that end of Xe fractionation is also separated from atmospheric H_2_ evolution, instead presumably being linked to declining bombardment flux or a waning of the martian magnetic field. The latter scenario cannot explain when Xe fractionation ceases, because ALH84001, which records a Xe isotopic composition close to the modern martian atmosphere also preserves a magnetic field (*11*, *13*). However, this serves to emphasize that for both the model we propose and those previously considered (*11*), the presence of a martian magnetic field is critical in guiding ionized Xe from the planet while leaving non-ionized gases behind. Future insights into the history of the martian dynamo will therefore provide tests of these models of Xe escape.

A key geological prediction impact-driven Xe fractionation makes is of the mass of late delivery to Mars. Estimates from HSE abundances in Mars’ mantle, inferred from martian meteorites, place the mass of material delivered postcore closure to be ∼0.4 to 1% of the mass of the planet (*61*, *62*). Figure 5C calculates the integrated mass delivered over time for the median solution (red line) and a sample of the posteriors (light orange). Many of these solutions fall above the window of permissible mass delivery; however, a significant fraction (∼45%) have parameters governing the loss and fractionation of Xe that allow for smaller impactor mass fluxes. Impact-driven loss is, therefore, in principle, able to operate within the independent constraints on mass delivery to Mars to explain the history of its atmospheric Xe mass fractionation.

We can look more closely at the properties of those simulations that successfully match the mass delivery constraint to understand the broader requirements for conditions in the early solar system and on Mars if impact bombardment is to explain the Xe fractionation. Figure 6 highlights the posterior distributions for the mass accreted to Mars over the early period of intense bombardment (*M*_accreted_), the timing of the impact peak (*t*_peak_), and the atmospheric surface pressure during the bombardment interval (*p*_surf_). For models accreting <1% the mass of Mars (∼45% of simulations), in line with the HSE observations, the posteriors are shown in dark orange. The key insight from these posterior distributions is that a narrow timing of the onset of Xe fractionation and lower atmospheric pressure on Mars (<1 bar) is favored for successful model runs. The reasons for these parameter values being favored are that (i) too early an onset of atmospheric escape leads to too much Xe isotopic fractionation occurring before the ∼4.4 Ga NWA11220-derived constraint on martian atmospheric Xe (*11*), and (ii) increased atmospheric pressure suppresses the degree of Xe fractionation observed, as a smaller fraction of the initial Xe pool ends up processed by impacts. Combined, these aspects of the impact-driven loss scenario favor Mars’ atmosphere beginning to record fractionation from ∼200 Myr after the birth of the solar system, with a low pressure atmosphere at this time.

Early peaks in impactor rate have long been predicted by dynamical models seeking to match the architecture of the solar system (*25*, *63*). However, there has been much debate over the timing of such events. Measurements of Xe isotopes in martian meteorites NWA11220 and ALH84001, dated at ∼4.4 and 4.16 Ga, respectively, tightly constrain the timing of impact-driven fractionation. Between these two measurements, mass fractionation of Xe isotopes increases from ∼1.6 to ∼4.4%/amu, essentially the modern value of the Mars atmosphere (*6*, *10*, *11*). In our simplified model, this epoch of Xe fractionation must be matched directly by a peak of impact bombardment. However, more complex models that couple a time evolving atmospheric mass with monotonically declining impactor flux [an “accretion tail” scenario (*26*)] could likely also be reconciled to the data: Such models have been previously suggested for Mars’ atmospheric evolution (*36*, *64*). In light of our impact-based description of the Xe isotope data, accretion-tail scenarios would need to be paired with an atmospheric mass stabilizing at ∼4.35 Ga to an initial solar composition from which isotopic fractionation could in-grow.

Even if in principle the model cannot separate an accretion tail scenario from am impact peak, our results do place an upper limit on how long after solar system birth any giant planet instability and associated bombardment flux can occur. Xe fractionation, as well as therefore intense impact sculpting of the atmosphere, needs to end by 300 to 400 Myr after CAI (calcium-aluminium-rich inclusion) formation, else martian Xe would experience a more protracted period of isotopic evolution than it exhibits. This is an important bound on the timing of giant planet instability, given the difficulty of directly probing impact history this far back in time (*65*, *66*).

An important feature of impact-driven Xe loss and its relation to Mars’ impact chronology is that it is most efficient for small impactors, those just above the threshold to eject atmosphere (*37*). Larger impactors, such as those responsible for large-scale basin formation (e.g., Hellas basin), are both less efficient at removing atmosphere per unit mass and eject all atmosphere above the tangent plane to the planet (*37*), thereby being less efficient at fractionating the atmosphere. Whereas it is the differential loss of atmosphere that is possible with smaller impactors that drives Xe fractionation. In this sense, Mars’ history of Xe fractionation, if driven by impacts, is a complementary archive of impact bombardment to the surface geological record: Whereas the latter best preserves and age dates (*67*, *68*) the large events, the former integrates the effects of the much more numerous small events. How these large basin-forming impactors could have affected atmospheric Xe is through remelting of the crust and mantle, processes which may release trapped Xe. A late (i.e., young) age of these large basin-forming events may help explain a peculiar feature of the martian Xe isotopic record: That in our quantification of the Xe fractionation (Fig. 5A), the martian meteorites record a peak fractionation at ALH84001 followed by a decline between then and the modern atmosphere (and, albeit with lower certainty, Nahkla). Large impacts liberating Xe from the martian mantle, or delivering some solar-like Xe, could explain this slight reversal in fractionation trend.

An impact solution to Mars’ fractionated Xe also has implications for its climate. Our results favor a low pressure atmosphere, <1 bar, remaining after formation and impact bombardment (Fig. 6C). This result is consistent with independent constraints on Mars’ early atmospheric pressure (*64*, *69*) and the observation of sulfur mass–independent isotopic fractionation in martian meteorite NWA11220 (*70*, *71*), which favors low atmospheric pressures. A tenuous atmosphere is consistent with a climate history of punctuated warmth and episodes of surface liquid water, with the later tail of impacts contributing to this stochasticity (*14*).

Impact-driven loss and fractionation of Xe may also be tested by NASA’s planned DAVINCI (Deep Atmosphere Venus Investigation of Noble gases, Chemistry, and Imaging) mission to Venus (*72*), which has as a core science aim measurement of the heavy noble gases in the planet’s atmosphere. If modern Venus is representative of the planet’s past, then its Xe isotopes should not show preferential fractionation compared to Kr, and the Kr to Xe ratio should be subchondritic rather than superchondritic as on Mars. This is because Venus’s massive atmosphere would stifle the efficiency of impact-driven fractionation of Xe: First, we have shown that for a thick atmosphere, too little Xe is removed to perturb the existing inventory; and second, both the model presented here and that in (*12*) require a magnetic field to channel ions away from the planet, the thick atmosphere and resulting hot surface and slow mantle cooling may have suppressed Venus’s dynamo for its entire history preventing this process. Conversely, in this paradigm, if DAVINCI’s measurements do evidence preferential Xe loss and fractionation, then it may point to a more temperate early Venus [e.g., as propsed (*73*)].

The history of Xe on Mars has importance for Earth’s own Xe depletion. Our experiments and calculations show the potential of impactors to fractionate Xe early in a planet’s history. This impact-driven fractionation occurs too early to explain the slow ingrowth of Xe mass fractionation seen in Earth’s atmosphere (*4*). However, recent results have shown the presence of a more ancient history of Xe fractionation in Earth’s deep mantle (*15*). This signal may point to Earth having once experienced similar impact loss processes to Mars, albeit resolvable only in mantle long hidden from subsequent volatile addition and recycling.

## MATERIALS AND METHODS

### Apparatus

The plasma UV-visible measurements were performed inside a vacuum-sealed cylindrical glass cell. The sample was contained within the cell using three diagnostic quartz windows and an in-house SwagelokTM gas-vacuum handling system. The Nd:YAG 1064-nm laser, capable of delivering a maximum energy of 850 mJ, was used to generate a plasma spark at the vessel’s center. This laser radiation was focused using a coated plano-convex quartz lens (= 1.5 cm, *f* = 10.5 cm). The emission spectra of the laser-induced plasma were then captured by the ESA 4000 Echelle spectrograph (LLA Instruments GmbH, Germany) using a fiber optic cable. A photodiode was attached to detect the laser spark radiation.

The laser pulses were cumulated at a repetition frequency of 10 Hz to simulate an impact shock wave. The observation gate delay was adjusted to varying values to facilitate time-resolved screening while maintaining a constant gate width of 500 ns. The time gating was controlled using ESAWIN software (version 14.3.0).

A vacuum pump and pressure gauge from Pfeiffer Vacuum Austria GmbH were used to manage the gas flow and measure pressure within the cell. A schematic of the experimental setup can be found in the Supplementary Materials (fig. S1).

### Experimental conditions

The vessel was filled with pure noble gases and their mixtures under a series of nominal pressures and concentrations investigated in this study: pressures of 40 to 700 torr and hydrogen concentration ranges from 0 up to 90%. For any measurements, certified gas samples, i.e., respectively 5.6 Linde Gas argon, 5.0 Linde Gas xenon, and 6.0 Linde Gas dihydrogen were used. The spectra have been recorded by an Echelle spectrograph with delays of 10, 500, 1000, 1500, 2000, 2500, and 3000 ns with the gate width of 100 ns.

### Experimental data acquisition and processing

Approximately 250 spectra were recorded in the wavelength range between 200 and 750 nm. A simple preprocessing procedure (*47*) was applied to all spectra to obtain basic plasma diagnostics. This analysis was performed by in-house programmed scripts in python-numpy and python-scipy. Theoretical values for the calculations were extracted from the National Institute of Standards and Technology database (*74*).

## Supplementary Materials

### This PDF file includes:

Supplementary Text Figs. S1 to S6 Table S1 References
