# Supplementary material for: Impact sculpting of the early martian atmosphere
Source: Sci Adv. 2024 Sep 11;10(37):eadm9921. doi: 10.1126/sciadv.adm9921 (PMC11639144; doi:10.1126/sciadv.adm9921)
Supplement: Supplementary file 1 — Supplementary Text Figs. S1 to S6 Table S1 References [file sciadv.adm9921_sm.pdf]

Science Advances
AAAS

Supplementary Materials for

Impact sculpting of the early martian atmosphere

Oliver Shorttle *et al.*

Corresponding author: Oliver Shorttle, shorttle@ast.cam.ac.uk

Sci. Adv. **10**, eadm9921 (2024)
DOI: 10.1126/sciadv.adm9921

This PDF file includes:

Supplementary Text
Figs. S1 to S6
Table S1
References

Supplementary Material

Here we provide supplementary information of the article: "Impact sculpting of the early Martian atmosphere":

S1:Experimental Setup

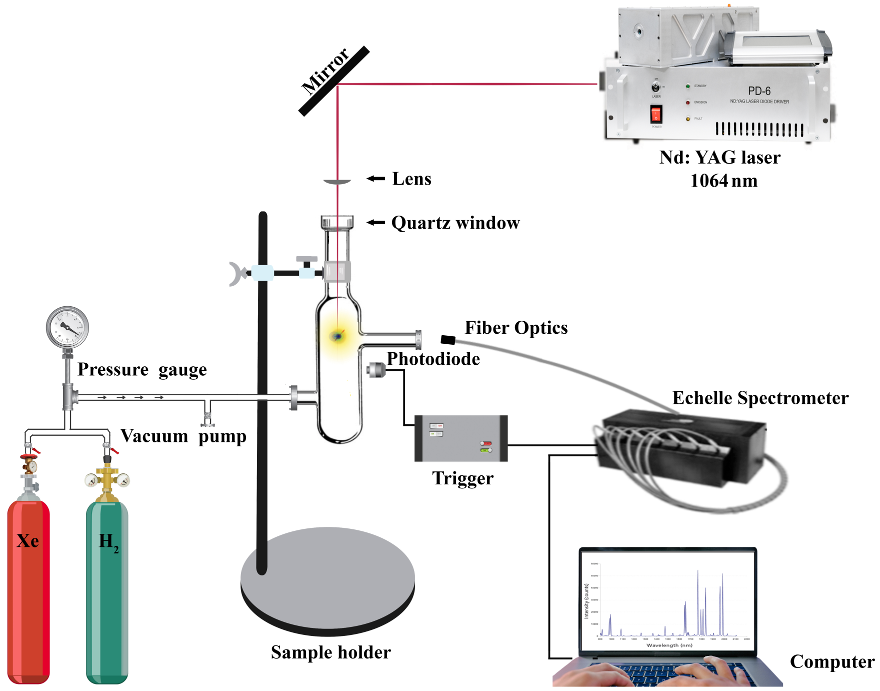

Figure S1. Schematic of the experimental setup for laser ionisation of gases.

S2: pre-processing procedure

First, certain transition lines observable under our conditions in all the experiments were selected. Then, by fitting the experimental data with the Lorentzian or/and Voigt function with in-house programmed scripts in PYTHON-NUMPY, the FWHM of this line is obtained. The initial analyses of the recorded spectra show that only the single-ionized states of Xe be visible in all records. The spectrum of the studied gaseous systems at different temperatures was simulated, thereby finding atomic and higher ionic lines very weak in our our experimental conditions. It is worth highlighting, however, that no Einstein coefficients for Xe^{2+} lines are, to the best knowledge of the authors, available *via* NIST or other reliable databases. Hence, in the Xenon simulation spectra, the Xe^{2+} effect had to be neglected. We must note, nonetheless, that the emission strength itself does not have to unequivocally report on the species abundance desired by further analyses. The results of those analyses brought out an evaluation of the excitation temperature, electron density and population of excited states, i.e., respectively $Xe^{\star}$ to Xe^{3+} and $Ar^{\star}$ to Ar^{3+} relative abundances in both pure and mixture plasma, as presented in Fig. S2, and Fig. S3. To calculate the excitation temperature and ratios for different ionization states, well-defined CF-LIBS spectral analysis (For more information see (*79*)) and its numerical interpretations were used.

With such qualitative results indicating on several boundary conditions for an independent numerical procedure, the CF-LIBS-based evaluation of the experimental diagnostics was appended by numerical simulations based on both line profile fitting and an overall LTE model. For their needs, all possible energy levels and the respective spectral lines were set as an initial basis. Their line profiles and the simulated spectra were changed iteratively until a statistically relevant agreement with the experimental data had been reached. The standard manual processing of displayed data

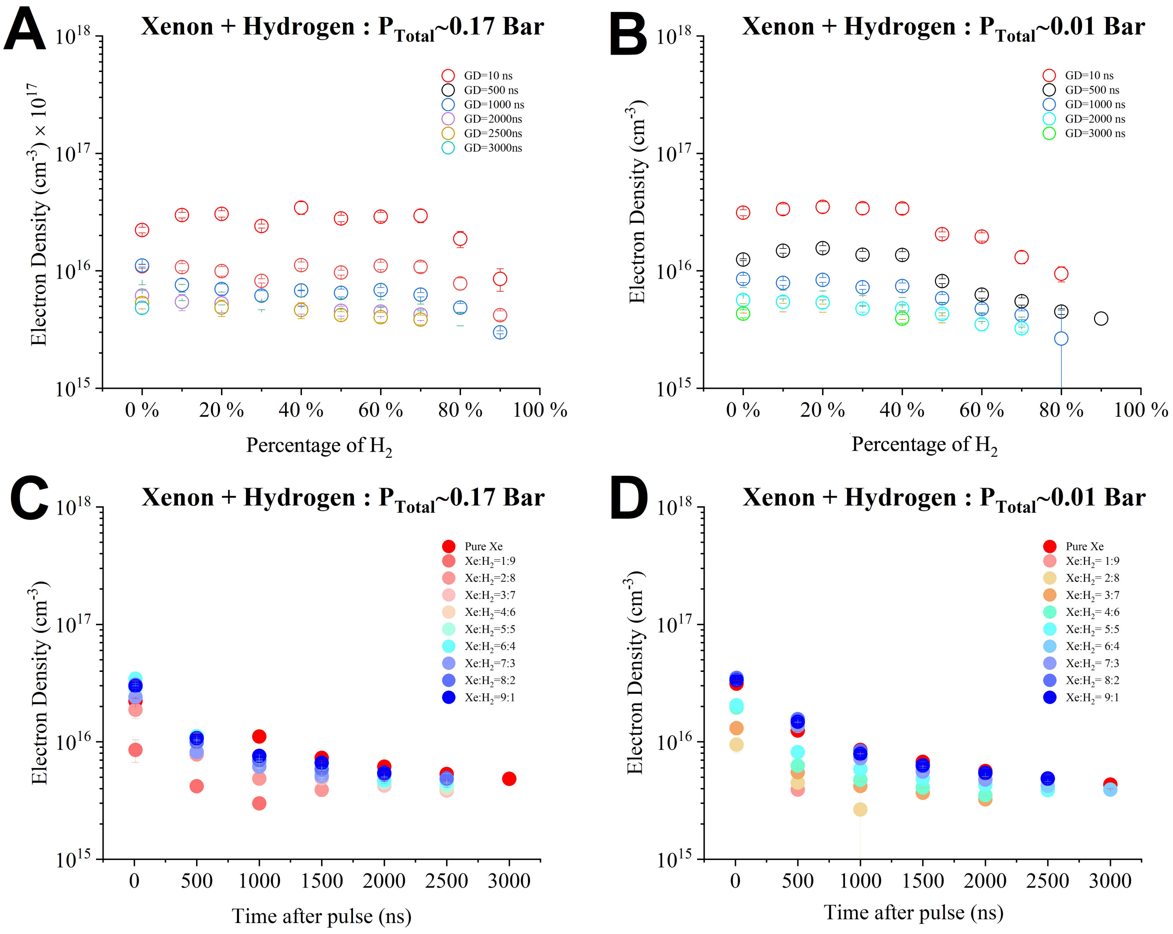

Figure S2. Electron density time-series in Xe ionisation experiments. (A-B) Show the electron density as a function of the percentage of H_2 in the gas mixture for total pressure 75 and 125 Torr, respectively. Each color corresponds to the specific time series. (C-D) represent time evolution of electron density during the plasma decay at different Xe:H_2 ratio for total pressure of 72 and 120 Torr, respectively.

therefore yet served as a control procedure for the comprehensive numerical study. In the modelling, we focus on the abundances of all species of theoretical interest, i.e., free electrons and all ionization states of noble gases considered, respectively $Xe^{\star}$ to Xe^{3+} and $Ar^{\star}$ to Ar^{3+}.

S3: Xe loss and fractionation modelling

Xe loss and fractionation modelling was performed by connecting the new experimental results with a simple model of Xe lofting through the atmosphere following impact, and its time-dependent recombination. Any ionized Xe reaching the homopause was then considered lost.

Electron Recombination of Ionized Xenon

At a very short time after the plasma is initiated, virtually all of the Xenon is ionized. It is not feasible to observe the plasma over this short timescale; the plasma is observed microseconds after the initiation. For pure Xenon, the electron recombination rate can be assessed by solving the following differential equation:

$$\frac{\mathrm{d}n(\mathrm{Xe})}{\mathrm{d}t} = -k_r n_e n(\mathrm{Xe}), \tag{S1}$$

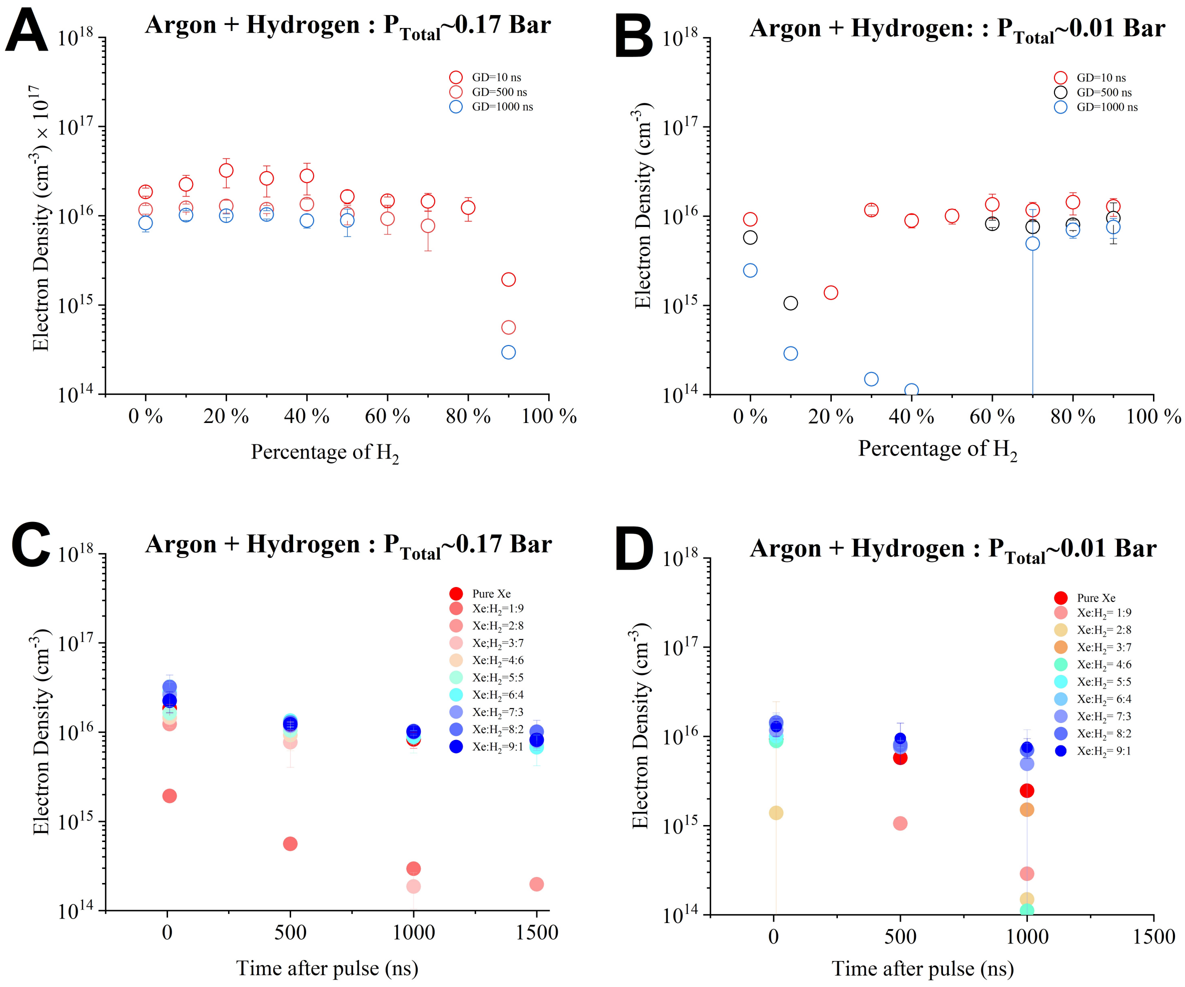

Figure S3. Electron density time-series in Ar ionisation experiments. (A-B) Show the electron density as a function of the percentage of H_2 in the gas mixture for total pressure 75 and 125 Torr, respectively. Each color corresponds to the specific time series. (C-D) represent time evolution of electron density during the plasma decay at different Ar:H_2 ratio for total pressure of 72 and 120 Torr, respectively.

where k_r [cm^3 s^{-1}] is the effective rate constant, and $n(\text{Xe})$ [cm^{-3}] and n_e [cm^{-3}] are number densities of Xe and electrons, respectively. We make the simplifying assumption that all the Xenon starts out singly ionized, and therefore that $n(\text{Xe}) = n_e$, in which case the equation has a simple solution (*80*):

$$n_e(t) = \frac{n_e(0)}{1 + n_e(0) k_r t}. \tag{S2}$$

We fit the t, n_e data from Section S2 for pure Xenon, and find the best fit is:

$$k_{r,\text{Xe}} = (6.0 \pm 1.0) \times 10^{-11}\ \text{cm}^3\,\text{s}^{-1}. \tag{S3}$$

The fit with data is shown in Figure S4.

It is important to note that this is the experimentally-measured effective rate constant, determined by the change in the number density of electrons over time. In reality, Xenon will be in multiple excited states, and multiply ionized, and

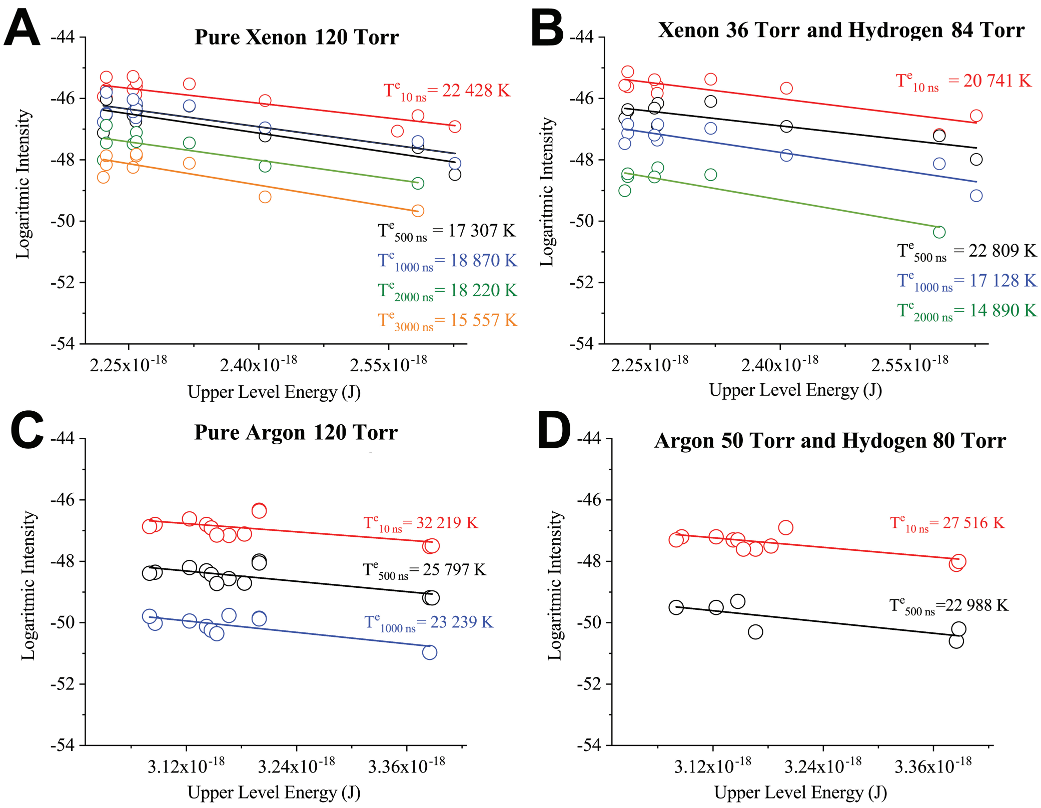

Figure S4. Fits to the recombination rate for Xe and Ar. (A–D) Depict the Boltzmann diagrams of pure Xenon/Argon and Xenon/Argon mixture with H_2 measured by Nd:YAG laser at p=120 Torr for Xe/Ar II, respectively. The spectral data measurement is performed at different delay times; 10, 500, 1500, 2000, 2500 and 3000 ns.

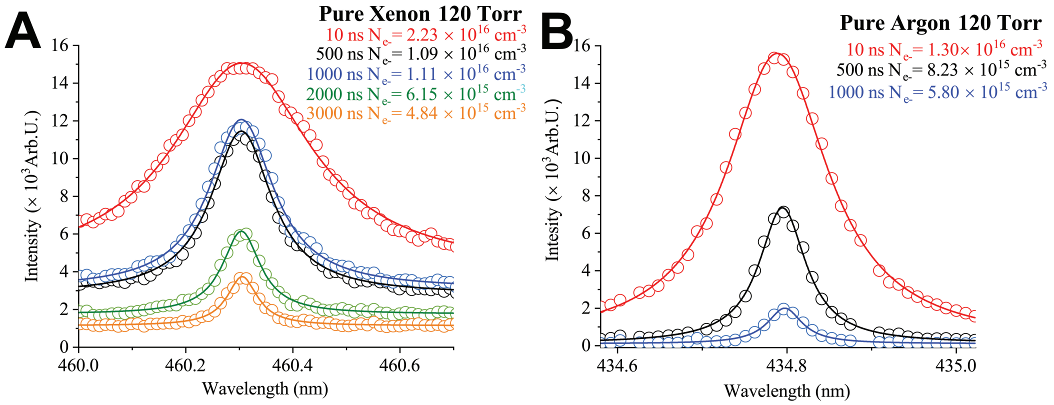

Figure S5. The Lorentzian fit for varying gate delay time of xenon/argon obtained using Nd:YAG laser.

many mechanisms are at play, such as collisional ionization, dissociative recombination with clusters of ionized Xenon (e.g. Xe_3^+, (*80*)), radiative association, among several others. These details are provided by Saeidfirozeh et a1. (47). Given that the plasma generated is analogous to an impact-generated plasma, the effective rate constant encapsulates all of these mechanisms in a regime appropriate to the Xenon loss mechanism we consider.

Argon can be treated similarly, using Eq. (S2) to fit and finding:

$$k_{r,\mathrm{Ar}} = \left(4.0 \pm 2.5\right) \times 10^{-10}\ \mathrm{cm^3\,s^{-1}}. \tag{S4}$$

The rate constant here has much larger error because the timescale is much shorter and difficult to accurately resolve with our experimental techniques. Fortunately, unlike with Xenon, we can compare with literature of effective recombination rate constants for Argon. For Xenon, the effective rate-constant is also determined to be second-order, as we find, but only an upper-limit was discovered .

We were able to find three values for the effective rate constant of Argon in the literature, two theoretical and one experimental. The theoretical values depend on detailed balance of energy states, including the continuum state for electrons, and differ due to differing calculations of cross-sections. Kelly (*81*), use their own calculation for electron-electron-ion cross-sections and Petschek & Byron's values for electron-netural-ion cross-sections (*82*). Hoffert (*83*), used the updated cross-section calculations of Zel'dovich & Raizer (*84*), and found a significantly higher value 2.83 times that of Kelly (*81*). Experiments by Owano et al. (*85*), found values 2.5 times that of Kelly (*81*). Each of these rate constants was presented as third-order rate constant in the literature; we converted these to second-order rate constants by multiplying the third-order constants by $\left(1\,\mathrm{bar}\right)/kT$ and then comparing the values at room temperature. The experimental results present the rate constant multiplied by a Boltzmann factor to estimate the ratio of ions to neutrals (*85*); to compare to our effective rate constants, we must neglect this factor. We find the rate constants of $1.29 \times 10^{-10}\ \mathrm{cm^3\,s^{-1}}$ for Kelly (*81*), $3.59 \times 10^{-10}\ \mathrm{cm^3\,s^{-1}}$ for Hoffert (*83*), and $\left(4 \pm 1\right) \times 10^{-10}\ \mathrm{cm^3\,s^{-1}}$ for Owano et al. (*85*). We estimate error bars for the experiment based on the quality of the fit with measured data, but no error was given for the theoretical calculations.

For Xenon, we find two values, one an upper limit of $10^{-9}\ \mathrm{cm^3\,s^{-1}}$ (46), and the other an estimate of $2.58 \times 10^{-12}\ \mathrm{cm^3\,s^{-1}}$ from Zahnle et al. (*12*), for radiative association of Xe^{+} with its electron, which can be treated as a lower-limit for the recombination rate. We present the effective rate constants from the literature along with the rate constants we measured in Figure S6.

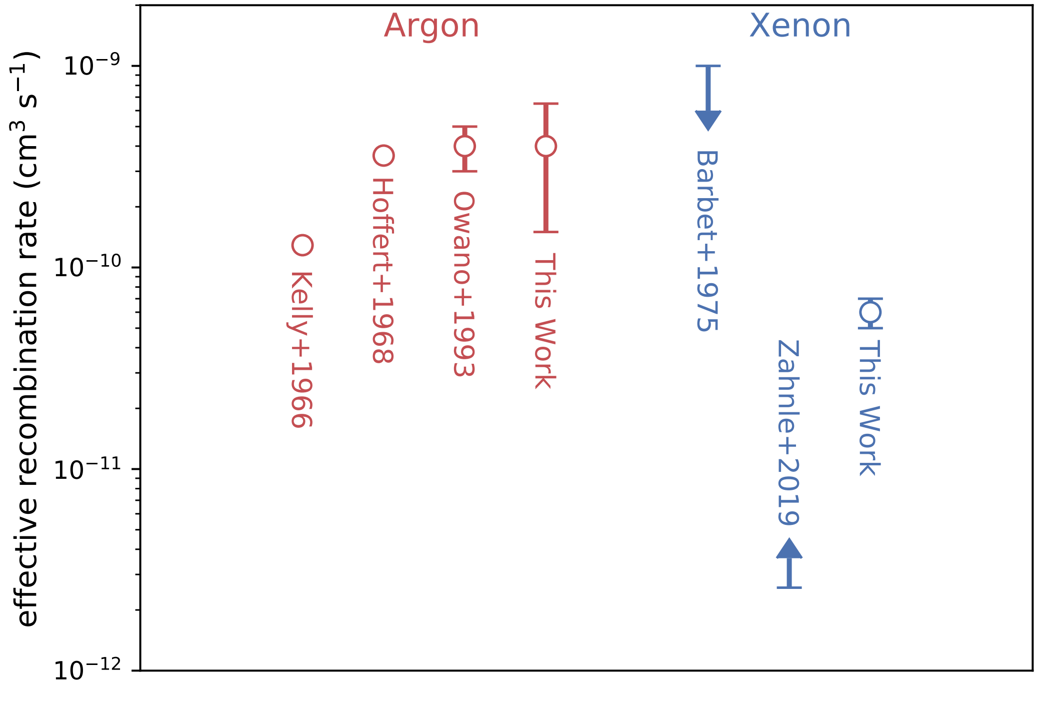

Figure S6. Values of the effective second-order rate coefficient for ion-electron recombination of both Xenon and Argon. The y-axis units are $\mathrm{cm^3\ s^{-1}}$. Data are from refs. *(81, 85, 46, 12)*.

Calculation of Xe loss and fractionation

We first consider the velocity, $v(h)$, needed to liberate a Xenon atom from some height in the atmosphere z to another height z' where $h = z' - z$ (km). Here we stipulate that z and z' are both $\ll R_p$, where R_p (km) is the radius of the planet. The velocity needed to move Xe over a distance h is found by balancing kinetic and gravitational potential energy. The Xenon will travel a length of time, t [s] to traverse a distance h, and:

$$t = \sqrt{\frac{2h}{g}}, \tag{S5}$$

where g [m s^{-2}] is the surface gravitational acceleration. We apply this velocity to the analysis of (37), momenta are balanced, and the momentum ejected by an impact is proportional to the momentum of the impactor (where $m_{\rm imp}$ [kg] is impactor mass and $v_{\rm imp}$ [m/s] is impactor velocity, $\mathscr{M}_{\rm esc}$ [kg] is the mass of the atmosphere that escapes and $v_{\rm esc} = \sqrt{2gR_p}$ [m/s] is the escape velocity):

$$m_{\rm imp} v_{\rm imp} \propto \mathscr{M}_{\rm esc} v_{\rm esc}, \tag{S6}$$

and (37) assume that $v_{\rm imp}$ is on the order of $v_{\rm esc}$. We assume the same for $v_{\rm imp}$ but additionally replace $v_{\rm esc}$ with $v(h)$ on the RHS of Eq. (S6), and $\mathscr{M}_{\rm esc}$ with $\mathscr{M}_{\rm th}$ [kg], the mass of the atmosphere removed above the homopause:

$$m_{\rm imp} \propto \mathscr{M}_{\rm th} \frac{v(h)}{v_{\rm esc}} = \mathscr{M}_{\rm th} \sqrt{\frac{h}{R_p}}, \tag{S7}$$

and find that the minimum impactor mass following (37), with the average surface atmospheric density $\rho_{\rm atm} = \mu_a m_p p/(kT)$, and p (bar) is the pressure at height z, k is Boltzmann's Constant, T (K) is the temperature of the atmosphere at height z, μ_a is the mean molecular weight of the atmosphere, and m_p is the mass of a proton:

$$m_{\rm min} = 4\pi \rho_{\rm atm} \left(\frac{kT}{\mu_a m_p g}\right)^3 \sqrt{\frac{h}{R_p}}, \tag{S8}$$

and therefore the minimum impactor radius, $r_{\rm min}$ [km] to liberate to z' the atmosphere directly above to be:

$$r_{\rm min} = \left(\frac{3\rho_{\rm atm}}{\rho_{\rm imp}}\right)^{1/3} \left(\frac{h}{R_p}\right)^{1/6} \frac{kT}{\mu_a m_p g}, \tag{S9}$$

where $\rho_{\rm imp}$ [kg m^{-3}] is the density of the impacting object, which we take to be 2000 kg m^{-3}. The atmospheric mass that is delivered to height z' is then (following (37)):

$$\mathscr{M}_{\rm th} = \frac{r_{\rm min}}{2r_{\rm imp}} \left[1 - \left(\frac{r_{\rm min}}{r_{\rm imp}}\right)^2\right] m_{\rm imp}, \tag{S10}$$

$$= \frac{2}{3}\pi r_{\rm imp}^2 r_{\rm min} \rho_{\rm imp} \left[1 - \left(\frac{r_{\rm min}}{r_{\rm imp}}\right)^2\right]. \tag{S11}$$

We apply Eq. (S9) to Eq. (S11) to find:

$$\mathscr{M}_{\rm th} = \frac{2}{3}\pi \left(\frac{3\mu_a m_p \rho_{\rm imp}^2 p}{kT}\right)^{1/3} \left(\frac{h}{R_p}\right)^{1/6} \frac{kT}{\mu_a m_p g} \times \left[r_{\rm imp}^2 - \left(\frac{3\mu_a m_p p}{\rho_{\rm imp} kT}\right)^{2/3} \left(\frac{h}{R_p}\right)^{1/3} \left(\frac{kT}{\mu_a m_p g}\right)^2\right]. \tag{S12}$$

What we are interested in is the mass of the ionized Xenon and Argon that reach the height z'. We will assume that the ionized Xenon and Argon travel a distance h with a time of flight inversely proportional to the square root of their mean molecular weights, either that they are mass segregated in the shock front through their time of flight or that the heavier ions spend more time in the proximity of the magnetic field before they escape, and therefore have more time to become neutralized and then settle back into the lower atmosphere. We set the time of the flight to:

$$t_{\mathrm{fl}} = \sqrt{\frac{h}{2g}}\sqrt{\frac{\mu(\mathrm{X})}{\mu_a}}, \tag{S13}$$

where $\mu(\mathrm{X})$ is the mean molecular mass of species X, either Ar or Xe. The ions go through two phases. First, they are caught up in the plasma shock, which disperses after $\sim 10\ \mu$s (*86*). During this time, the number density of species X, $n(\mathrm{X})$ cm^{-3} follows the solution to Eq. (1) under the assumption that $n_e \propto n(\mathrm{X})$. After the plasma disperses, the electron number density is set by other processes, XUV photoionization or SEP (solar energetic particle) ionization, and n_e can be treated as a constant, whereupon Eq. (1) is a linear differential equation with the solution:

$$n(\mathrm{X}^+) = n_0(\mathrm{X}^+)\, e^{-k_r n_e t_{\mathrm{fl}}}. \tag{S14}$$

Here, $n_0(\mathrm{X}^+)$ (cm^{-3}) is the number density of X^+ after the plasma disperses, and $n(\mathrm{X}^+)$ (cm^{-3}) is the number density of X^+ at the time it reaches the height h, and k_r (cm^3 s^{-1}) is the experimentally measured electron-ion recombination constant. It is useful to define the mass mixing ratio $f_m(\mathrm{X})$ which is related to the volume mixing ratio as $f_m(\mathrm{X}) = \mu(\mathrm{X}) f(\mathrm{X})/\mu_a = \mu(\mathrm{X}) n(\mathrm{X})/(\mu_a n_{\mathrm{gas}})$, where n_{gas} (cm^{-3}) is the total number density of the gas at height z. It is also useful to define the ratio of the ionized over the total amount of species X, $\chi(\mathrm{X}^+)$. On this basis, we can define the total mass of X^+ that reaches height h as:

$$\mathscr{M}(\mathrm{X}^+) = f(\mathrm{X})\chi_0(\mathrm{X}^+)\left(\frac{\mu(\mathrm{X})}{\mu_a}\right)\mathscr{M}_{\mathrm{th}} \exp\left\{-k_r n_e \sqrt{\frac{h}{2g}}\sqrt{\frac{\mu(\mathrm{X})}{\mu_a}}\right\}. \tag{S15}$$

We can cast Eq's (S12) and (S15) in more useful compact terms:

$$\mathscr{M}(\mathrm{X}^+) = \left(\frac{\mu(\mathrm{X})}{\mu_a}\right) f(\mathrm{X})\chi_0(\mathrm{X}^+) e^{-\Lambda_r}\, \rho_{\mathrm{imp}} R_0^3 \left[\left(\frac{r_{\mathrm{imp}}}{R_0}\right)^2 - 1\right], \tag{S16}$$

where the radius:

$$R_0 = \left(\frac{3\mu_a m_p p}{\rho_{\mathrm{imp}} kT}\right)^{1/3}\left(\frac{h}{R_p}\right)^{1/6}\left(\frac{kT}{\mu_a m_p g}\right). \tag{S17}$$

And the recombination scale:

$$\Lambda_r = f_e k_r \frac{p}{kT}\sqrt{\frac{h}{2g}}\sqrt{\frac{\mu(\mathrm{X})}{\mu_a}}. \tag{S18}$$

This is the effect of a single impact. To calculate the cumulative effect of impacts on Xe and Ar depletion, we need to apply an impact history, which we will represent by a power law:

$$dN = \beta N_0 \left(\frac{R_0}{r_{\mathrm{imp}}}\right)^{\beta} \frac{dr_{\mathrm{imp}}}{r_{\mathrm{imp}}}, \tag{S19}$$

where $\beta > 2$ is a constant, r_{imp} (km) is the impactor radius, and N_0 is the number of impactors at $r_{\mathrm{imp}} = R_0$. We can integrate Eq. (S16) over dN to determine the total mass loss of Xe and Ar:

$$\mathscr{M}_D(\mathrm{X}) = \int_{R_0}^{\infty} \mathscr{M}(\mathrm{X}^+)\, dN. \tag{S20}$$

Solving the integral yields:

$$\mathscr{M}_D(\mathrm{X}) = \frac{2N_0}{\beta - 2}\left(\frac{\mu(\mathrm{X})}{\mu_a}\right) f(\mathrm{X})\chi_0(\mathrm{X}^+)e^{-\Lambda_r}\rho_{\mathrm{imp}}R_0^3. \tag{S21}$$

This equation is only valid as written if the depletion is small compared to the total mass of the Ar or Xe (where $f_0(\mathrm{X})$ is the initial mixing ratio of Xe or Ar):

$$\mathscr{M}_T(\mathrm{X}) = \frac{\mu(\mathrm{X})p_0 4\pi R_p^2}{\mu_a g} f_0(\mathrm{X}), \tag{S22}$$

where p_0 (bar) is the surface pressure. This is because, if $\mathscr{M}_D(\mathrm{X}) \sim \mathscr{M}_T(\mathrm{X})$ then $f(\mathrm{X})$ in Eq. (S21) is no longer constant. We can relate $f(\mathrm{X})$ to $f_0(\mathrm{X})$ in the form:

$$\mathscr{M}_D(\mathrm{X}) = \frac{2N_0}{\beta - 2}\left(\frac{\mu(\mathrm{X})}{\mu_a}\right) f_0(\mathrm{X})\left[\frac{\mathscr{M}_T(\mathrm{X}) - \mathscr{M}_D(\mathrm{X})}{\mathscr{M}_T(\mathrm{X})}\right]\chi_0(\mathrm{X}^+)e^{-\Lambda_r}\rho_{\mathrm{imp}}R_0^3, \tag{S23}$$

and then solve for $\mathscr{M}_D(\mathrm{X})$ to find:

$$\mathscr{M}_D(\mathrm{X}) = \frac{\mathscr{M}_0(\mathrm{X})f_0(\mathrm{X})\mathscr{M}_T(\mathrm{X})}{\mathscr{M}_0(\mathrm{X})f_0(\mathrm{X}) + \mathscr{M}_T(\mathrm{X})}, \tag{S24}$$

where

$$\mathscr{M}_0(\mathrm{X}) = \frac{2N_0}{\beta - 2}\left(\frac{\mu(\mathrm{X})}{\mu_a}\right)\chi_0(\mathrm{X}^+)e^{-\Lambda_r}\rho_{\mathrm{imp}}R_0^3. \tag{S25}$$

The quantities R_0 and Λ_r can be parameterized in terms of the free and experimentally-constrained parameters given above.

$$\begin{aligned} R_0\,(\mathrm{km}) &= 28.45\,(\mu_a)^{1/3}\left(\frac{1\,\mathrm{g\,cm^{-3}}}{\rho_{\mathrm{imp}}}\right)^{1/3}\left(\frac{h}{1\,\mathrm{km}}\right)^{1/6}\left(\frac{R_\oplus}{R_p}\right)^{1/6}\left(\frac{1\,\mathrm{m\,s^{-2}}}{g}\right) \\ &\times\left(\frac{p}{1\,\mathrm{bar}}\right)^{1/3}\left(\frac{T}{300\,\mathrm{K}}\right)^{2/3} \end{aligned} \tag{S26}$$

$$\begin{aligned} \Lambda_r &= \left(5.4\times 10^{20}\,\mathrm{cm^{-3}\,s}\right) f_e k_r\left(\frac{p}{1\,\mathrm{bar}}\right)\left(\frac{300\,\mathrm{K}}{T}\right)\left(\frac{h}{1\,\mathrm{km}}\right)^{1/2} \\ &\times\left(\frac{1\,\mathrm{m\,s^{-2}}}{g}\right)^{1/2}\left(\frac{\mu(\mathrm{X})}{\mu_a}\right)^{1/2}. \end{aligned} \tag{S27}$$

We apply a simple prescription for the impact mass flux, of a simple spike of impacts, normally distributed in time, overlain upon a constant background. This spike, being narrow and timed early in the solar system's history, has the effect of creating an early high impact flux that peaks 1-3 orders of magnitude higher (depending on parameter choices) and then declines to lower levels after several hundred million years as frequently modelled (54). The impact flux equation takes the form of

$$\log_{10}\left\{\left(\frac{1}{1\,\mathrm{g\,y^{-1}}}\right)\frac{dm}{dt}\right\} = 13 + C_{\mathrm{peak}}\exp\left(-\frac{1}{2}\left(\frac{t - t_{\mathrm{peak}}}{\Delta t}\right)^2\right), \tag{S28}$$

where t is the time after the start of the solar system (Gyr), C_{peak} is a factor describing the magnitude of the transient increase in bombardment and Δt is the width of the impactor spike (Gyr). Mass is converted to N_0 by:

$$N_0 = \frac{3}{4\pi\rho_{\mathrm{imp}}R_0^3}\int_{t_0}^{t}\left(\frac{dm}{dt'}\right)dt', \tag{S29}$$

where t_0 (Ga) is the time where the ions start to be depleted from the atmosphere.

We will put the results of this calculation in terms of relative depletion factors and fractionation. The depletion factor for species X is:

$$D(\mathrm{X}) = \frac{\mathscr{M}_T(\mathrm{X})}{\mathscr{M}_T(\mathrm{X}) - \mathscr{M}_D(\mathrm{X})}, \tag{S30}$$

and results will be plotted in terms of relative depletion, or $D(\mathrm{Xe})/D(\mathrm{Ar})$, since either through impacts that remove the bulk atmosphere or other processes, both Xe and Ar, and all volatiles, are depleted relative to refractories. Xenon is further depleted relative to the other Nobel gases.

Fractionation, F, is represented in units of %/amu, by the equation:

$$F(\mathrm{X}_1, \mathrm{X}_2) = \frac{100\%}{|\mu(\mathrm{X}_1) - \mu(\mathrm{X}_2)|} \left(\frac{\mathscr{M}_T(\mathrm{X}_1) - \mathscr{M}_D(\mathrm{X}_1)}{\mathscr{M}_T(\mathrm{X}_2) - \mathscr{M}_D(\mathrm{X}_2)} \right) - 100\%. \tag{S31}$$

For Mars we take $R_p = 0.532 R_\oplus$ and $g = 3.71$ m s^{-2}. We consider an N_2 and CO_2 dominated atmosphere ($mu_a = 29.6$) with low surface pressure, $p_0 = 0.1$ bar, an isothermal atmosphere with temperature $T = 300$ K for simplicity, since the results are not very sensitive to temperature and are not significantly affected for 200 K $< T <$ 400 K. The initial mixing ratios of Ar and Xe are 7.2×10^{-3} and 5×10^{-7}, respectively. The impactor mass density is taken to be 2 g cm^{-3}, and for the power-law size distribution, $\beta = 2.1$.

We adjusted f_e and z to find the best fit and found the best results when the impactor liberates the atmosphere from $z = 40$ km ($p = 1$ mbar) to $z' = 140$ km ($h = 100$ km), and when the density-weighted average ionization fraction along the liberated atmosphere's trajectory, $f_e = 1.35 \times 10^{-8}$. The results are not too sensitive to z, permitting ranges of z from 20 to 100 km that still provide good fits. The results are very sensitive to f_e. When $z = 40$ km, then changes of 5×10^{-10} will move either the depletion or, more often, the fractionation, well outside error bars. There is a degeneracy between z, h and f_e, where smaller h or larger z require larger f_e to bring the results back into alignment with observations.

Table S1. **Symbols used in modelling Xe escape.**

Symbol	Definition
$k_r(\mathrm{Ar}) = 4.0 \pm 2.5 \times 10^{-10}$ cm^3 s^{-1}	electron-Ar^+ recombination rate coefficient
$k_r(\mathrm{Xe}) = 6.0 \pm 1.0 \times 10^{-11}$ cm^3 s^{-1}	electron-Xe^+ recombination rate coefficient
$\chi_0(\mathrm{Ar}) = 0.01$	ratio of Ar^+ to $Ar^\star$ after the plasma has dispersed
$\chi_0(\mathrm{Xe}) = 0.1$	ratio of Xe^+ to $Xe^\star$ after the plasma has dispersed
Physical constants	
$m_p = 1.673 \times 10^{-27}$ kg	mass of a proton
$m_e = 9.109 \times 10^{-31}$ kg	mass of an electron
$\mathrm{h} = 6.626 \times 10^{-34}$ J s	Planck's constant
$\mathrm{k}_B = 1.3806 \times 10^{-23}$ J K^{-1}	Boltzmann's constant
Free parameters	
Z	charge of a species
R_p (km)	radius of a planet
g (m s^{-2})	surface gravitational acceleration
p_0 (bar)	surface pressure
p (bar)	pressure at which small impactors remove Ar and Xe
T (K)	surface temperature
μ_a	mean molecular mass of the atmosphere
f_e	degree of ionization
$f(\mathrm{X})$	volume mixing ratio of species X
$\mu(\mathrm{X})$	molecular mass of species X
h (km)	height the ions need to reach in order to escape
r_{imp} (km)	impactor radius
ρ_{imp} (g cm^{-3})	impactor density

REFERENCES AND NOTES

1. R. O. Pepin, On the origin and early evolution of terrestrial planet atmospheres and meteoritic volatiles. *Icarus* **92**, 2–79 (1991).

2. M. Ozima, F. A. Podosek, *Noble Gas Geochemistry* (Cambridge Univ. Press, 2009).

3. M. Pujol, B. Marty, R. Burgess, Chondritic-like xenon trapped in archean rocks: A possible signature of the ancient atmosphere. *Earth Planet. Sci. Lett.* **308**, 298–306 (2011).

4. G. Avice, B. Marty, R. Burgess, A. Hofmann, P. Philippot, K. Zahnle, D. Zakharov, Evolution of atmospheric xenon and other noble gases inferred from archean to paleoproterozoic rocks. *Geochim. Cosmochim. Acta* **232**, 82–100 (2018).

5. L. Ardoin, M. Broadley, M. Almayrac, G. Avice, D. Byrne, A. Tarantola, A. Lepland, T. Saito, T. Komiya, T. Shibuya, B. Marty, The end of the isotopic evolution of atmospheric xenon. *Geochem. Perspect. Lett.* **20**, 43–47 (2022).

6. P. G. Conrad, C. A. Malespin, H. B. Franz, R. O. Pepin, M. G. Trainer, S. P. Schwenzer, S. Atreya, C. Freissinet, J. Jones, H. Manning, T. Owen, A. A. Pavlov, R. C. Weins, M. H. Wong, P. R. Mahaffy, In situ measurement of atmospheric krypton and xenon on mars with mars science laboratory. *Earth Planet. Sci. Lett.* **454**, 1–9 (2016).

7. T. D. Swindle, M. W. Caffee, C. M. Hohenberg, Xenon and other noble gases in shergottites. *Geochim. Cosmochim. Acta* **50**, 1001–1015 (1986).

8. R. O. Pepin, On the isotopic composition of primordial xenon in terrestrial planet atmospheres. *Space Sci. Rev.* **92**, 371–395 (2000).

9. B. Marty, K. Altwegg, H. Balsiger, A. Bar-Nun, D. Bekaert, J.-J. Berthelier, A. Bieler, C. Briois, U. Calmonte, M. Combi, J. De Keyser, B. Fiethe, S. A. Fuselier, S. Gasc, T. I. Gombosi, K. C. Hansen, M. Hässig, A. Jäckel, E. Kopp, A. Korth, L. Le Roy, U. Mall, O. Mousis, T. Owen, H. Rème, M. Rubin, T. Sèmon, C. Y. Tzou, J. H. Waite, P. Wurz, Xenon isotopes in 67p/churyumov-gerasimenko show that comets contributed to earth’s atmosphere. *Science* **356**, 1069–1072 (2017).

10. W. S. Cassata, Meteorite constraints on martian atmospheric loss and paleoclimate. *Earth Planet. Sci. Lett.* **479**, 322–329 (2017).

11. W. S. Cassata, K. J. Zahnle, K. M. Samperton, P. C. Stephenson, J. Wimpenny, Xenon isotope constraints on ancient martian atmospheric escape. *Earth Planet. Sci. Lett.* **580**, 117349 (2022).

12. K. J. Zahnle, M. Gacesa, D. C. Catling, Strange messenger: A new history of hydrogen on Earth, as told by Xenon. *Geochim. Cosmochim. Acta* **244**, 56–85 (2019).

13. B. P. Weiss, H. Vali, F. J. Baudenbacher, J. L. Kirschvink, S. T. Stewart, D. L. Shuster, Records of an ancient martian magnetic field in alh84001. *Earth Planet. Sci. Lett.* **201**, 449–463 (2002).

14. R. Wordsworth, A. H. Knoll, J. Hurowitz, M. Baum, B. L. Ehlmann, J. W. Head, K. Steakley, A coupled model of episodic warming, oxidation and geochemical transitions on early mars. *Nat. Geosci.* **14**, 127–132 (2021).

15. S. Péron, S. Mukhopadhyay, Pre-subduction mantle noble gas elemental pattern reveals larger missing xenon in the deep interior compared to the atmosphere. *Earth Planet. Sci. Lett.* **593**, 117655 (2022).

16. S. Mukhopadhyay, Early differentiation and volatile accretion recorded in deep-mantle neon and xenon. *Nature* **486**, 101–104 (2012).

17. D. M. Hunten, R. O. Pepin, J. C. Walker, Mass fractionation in hydrodynamic escape. *Icarus* **69**, 532–549 (1987).

18. K. J. Zahnle, Xenological constraints on the impact erosion of the early martian atmosphere. *J. Geophys. Res. Planets* **98**, 10899–10913 (1993).

19. T. J. Ahrens, Impact erosion of terrestrial planetary atmospheres. *Annu. Rev. Earth Planet. Sci.* **21**, 525–555 (1993).

20. V. S. Safronov, *Evolution of the Protoplanetary Cloud and Formation of the Earth and the Planets* (Keter Publishing House, 1972).

21. R. Greenberg, J. F. Wacker, W. K. Hartmann, C. R. Chapman, Planetesimals to planets: Numerical simulation of collisional evolution. *Icarus* **35**, 1–26 (1978).

22. D. C. Rubie, S. A. Jacobson, A. Morbidelli, D. P. O'Brien, E. D. Young, J. de Vries, F. Nimmo, H. Palme, D. J. Frost, Accretion and differentiation of the terrestrial planets with implications for the compositions of early-formed solar system bodies and accretion of water. *Icarus* **248**, 89–108 (2015).

23. M. Ćuk, S. T. Stewart, Making the moon from a fast-spinning earth: A giant impact followed by resonant despinning. *Science* **338**, 1047–1052 (2012).

24. B. P. Weiss, X.-N. Bai, R. R. Fu, History of the solar nebula from meteorite paleomagnetism. *Sci. Adv.* **7**, eaba5967 (2021).

25. R. Gomes, H. F. Levison, K. Tsiganis, A. Morbidelli, Origin of the cataclysmic late heavy bombardment period of the terrestrial planets. *Nature* **435**, 466–469 (2005).

26. G. Neukum, B. A. Ivanov, W. K. Hartmann, Cratering records in the inner solar system in relation to the lunar reference system. *Space Sci. Rev.* **96**, 55–86 (2001).

27. W. K. Hartmann, G. Neukum, Cratering chronology and the evolution of Mars. *Space Sci. Rev.* **96**, 165–194 (2001).

28. W. F. Bottke, H. F. Levison, D. Nesvorný, L. Dones, Can planetesimals left over from terrestrial planet formation produce the lunar late heavy bombardment? *Icarus* **190**, 203–223 (2007).

29. W. F. Bottke, R. J. Walker, J. M. Day, D. Nesvorny, L. Elkins-Tanton, Stochastic late accretion to earth, the moon, and mars. *Science* **330**, 1527–1530 (2010).

30. F. Tera, D. A. Papanastassiou, G. J. Wasserburg, Isotopic evidence for a terminal lunar cataclysm. *Earth Planet. Sci. Lett.* **22**, 1–21 (1974).

31. W. F. Bottke, M. D. Norman, The late heavy bombardment. *Annu. Rev. Earth Planet. Sci.* **45**, 619–647 (2017).

32. A. Morbidelli, D. Nesvorny, V. Laurenz, S. Marchi, D. C. Rubie, L. Elkins-Tanton, M. Wieczorek, S. Jacobson, The timeline of the lunar bombardment: Revisited. *Icarus* **305**, 262–276 (2018).

33. D. Nesvorný, Dynamical evolution of the early solar system. *Annu. Rev. Astron. Astrophys.* **56**, 137–174 (2018).

34. D. Nesvorný, D. Vokrouhlický, W. F. Bottke, H. F. Levison, Evidence for very early migration of the solar system planets from the patroclus–menoetius binary jupiter trojan. *Nat. Astron.* **2**, 878–882 (2018).

35. C. Avdellidou, M. Delbo', D. Nesvorný, K. J. Walsh, A. Morbidelli, Dating the solar system's giant planet orbital instability using enstatite meteorites. *Science* **348**, 348–352 (2024).

36. H. Melosh, A. Vickery, Impact erosion of the primordial atmosphere of mars. *Nature* **338**, 487–489 (1989).

37. H. E. Schlichting, R. Sari, A. Yalinewich, Atmospheric mass loss during planet formation: The importance of planetesimal impacts. *Icarus* **247**, 81–94 (2015).

38. H. E. Schlichting, S. Mukhopadhyay, Atmosphere impact losses. *Space Sci. Rev.* **214**, 34 (2018).

39. J. C. Walker, Impact erosion of planetary atmospheres. *Icarus* **68**, 87–98 (1986).

40. E. A. Silber, M. Boslough, W. K. Hocking, M. Gritsevich, R. W. Whitaker, Physics of meteor generated shock waves in the earth's atmosphere–a review. *Adv. Space Res.* **62**, 489–532 (2018).

41. A. Kramida, Yu. Ralchenko, J. Reader; NIST ASD Team, NIST Atomic Spectra Database (ver. 5.9), National Institute of Standards and Technology, Gaithersburg, *MD.* (2021); https://physics.nist.gov/asd (2022).

42. V. Anicich, A survey of bimolecular ion-molecule reactions for use in modeling the chemistry of planetary atmospheres, cometary comae, and interstellar clouds-1993 supplement. *Astrophys. J. Suppl. Ser.* **84**, 215–315 (1993).

43. H. S. Johnston, W. Kornegay, Rate and mechanism of the thermal ionization of xenon. *Trans. Faraday Soc.* **57**, 1563–1577 (1961).

44. W. M. Kornegay, H. S. Johnston, Kinetics of thermal ionization. II. Xenon and krypton. *J. Chem. Phys.* **38**, 2242–2247 (1963).

45. A. P. Vitols, H. J. Oskam, Electron-ion recombination of Xe^+ and XeH^+ ions. *Phys. Rev.* **8**, 3211–3214 (1973).

46. A. Barbet, N. Sadeghi, J. C. Pebay-Peyroula, Study of the electron-ion recombination processes in the xenon afterglow plasma. *J. Phys. B Atom. Mol Phys.* **8**, 1785–1794 (1975).

47. H. Saeidfirozeh, A. K. Myakalwar, P. Kubelík, A. Ghaderi, V. Laitl, L. Petera, P. B. Rimmer, O. Shorttle, A. N. Heays, A. Křivková, M. Krus, S. Civiš, J. Yáñez, E. Képeš, P. Pořízka, M. Ferus, ANN-LIBS analysis of mixture plasmas: Detection of xenon. *J. Anal. At. Spectrom.* **37**, 1815–1823 (2022).

48. K. Pahlevan, L. Schaefer, L. T. Elkins-Tanton, S. J. Desch, P. R. Buseck, A primordial atmospheric origin of hydrospheric deuterium enrichment on mars. *Earth Planet. Sci. Lett.* **595**, 117772 (2022).

49. F. Feroz, M. Hobson, M. Bridges, Multinest: An efficient and robust bayesian inference tool for cosmology and particle physics. *Mon. Not. R Astron. Soc.* **398**, 1601–1614 (2009).

50. F. Feroz, M. P. Hobson, E. Cameron, A. N. Pettitt, Importance nested sampling and the multinest algorithm. arXiv:1306.2144 [astro-ph.IM] (2013).

51. J. Buchner, A. Georgakakis, K. Nandra, L. Hsu, C. Rangel, M. Brightman, A. Merloni, M. Salvato, J. Donley, D. Kocevski, X-ray spectral modelling of the agn obscuring region in the cdfs: Bayesian model selection and catalogue. *Astron. Astrophys.* **564**, A125 (2014).

52. B. M. Jakosky, R. O. Pepin, R. E. Johnson, J. L. Fox, Mars atmospheric loss and isotopic fractionation by solar-wind-induced sputtering and photochemical escape. *Icarus* **111**, 271–288 (1994).

53. S. K. Atreya, M. G. Trainer, H. B. Franz, M. H. Wong, H. L. Manning, C. A. Malespin, P. R. Mahaffy, P. G. Conrad, A. E. Brunner, L. A. Leshin, J. H. Jones, C. R. Webster, T. C. Owen, R. O. Pepin, R. Navarro-González, Primordial argon isotope fractionation in the atmosphere of Mars measured by the sam instrument on curiosity and implications for atmospheric loss. *Geophys. Res. Lett.* **40**, 5605–5609 (2013).

54. S. Marchi, W. Bottke, L. Elkins-Tanton, M. Bierhaus, K. Wuennemann, A. Morbidelli, D. Kring, Widespread mixing and burial of earth's hadean crust by asteroid impacts. *Nature* **511**, 578–582 (2014).

55. C. A. Sinclair, M. C. Wyatt, A. Morbidelli, D. Nesvorný, Evolution of the Earth's atmosphere during late Veneer accretion. *Mon. Not. R. Astron. Soc.* **499**, 5334–5362 (2020).

56. P. Scherer, L. Schultz, Noble gas record, collisional history and pairing of CV, CO, CK and other carbonaceous chondrites *Meteorit. Planet. Sci.* **35**, 145–153 (2000).

57. N. Schelhaas, U. Ott, F. Begemann, Trapped noble gases in unequilibrated ordinary chondrites. *Geochim. Cosmochim. Acta* **54**, 2869–2882 (1990).

58. J. Crabb, E. Anders, Noble gases in E-chondrites. *Geochim. Cosmochim. Acta* **45**, 2443–2464 (1981).

59. N. Dauphas, T. Hopp, D. Nesvorný Bayesian inference on the isotopic building blocks of Mars and Earth. *Icarus* **408**, 115805 (2024).

60. M. R. Carroll, J. D. Webster, *Chapter 7. Solubilities of Sulfur, Noble Gases, Nitrogen, Chlorine and Fluorine in Magmas* (De Gruyter, 1994), pp. 231–280.

61. C. W. Dale, K. W. Burton, R. C. Greenwood, A. Gannoun, J. Wade, B. J. Wood, D. G. Pearson, Late accretion on the earliest planetesimals revealed by the highly siderophile elements. *Science* **336**, 72–75 (2012).

62. J. M. Day, R. J. Walker, Highly siderophile element depletion in the moon. *Earth Planet. Sci. Lett.* **423**, 114–124 (2015).

63. K. Tsiganis, R. Gomes, A. Morbidelli, H. F. Levison, Origin of the orbital architecture of the giant planets of the solar system. *Nature* **435**, 459–461 (2005).

64. M. Scherf, H. Lammer, Did Mars possess a dense atmosphere during the first ∼400 million years? *Space Sci. Rev.* **217**, 2 (2021).

65. M. D. Norman, The lunar cataclysm: Reality or "mythconception"? *Elements* **5**, 23–28 (2009).

66. C. R. Walton, H. Jeon, A. Černok, A. S. Rae, I. Baziotis, F. Tang, V. S. Kuppili, L. Ferrière, J. Darling, S. Hu, M. J. Whitehouse, M. Anand, O. Shorttle, In-situ phosphate U-pb ages of the L chondrites. *Geochim. Cosmochim. Acta* **359**, 191–204 (2023).

67. C. I. Fassett, J. W. Head, Sequence and timing of conditions on early mars. *Icarus* **211**, 1204–1214 (2011).

68. W. F. Bottke, J. C. Andrews-Hanna, A post-accretionary Lull in large impacts on early Mars. *Nat. Geosci.* **10**, 344–348 (2017).

69. E. S. Kite, J.-P. Williams, A. Lucas, O. Aharonson, Low palaeopressure of the martian atmosphere estimated from the size distribution of ancient craters. *Nat. Geosci.* **7**, 335–339 (2014).

70. Y. Endo, S. O. Danielache, Y. Ueno, Total pressure dependence of sulfur mass-independent fractionation by SO_2 photolysis. *Geophys. Res. Lett.* **46**, 483–491 (2019).

71. A. G. Tomkins, S. L. Alkemade, S. E. Nutku, N. R. Stephen, M. A. Finch, H. Jeon, A small S-MIF signal in martian regolith pyrite: Implications for the atmosphere. *Geochim. Cosmochim. Acta* **290**, 59–75 (2020).

72. L. S. Glaze, J. B. Garvin, B. Robertson, N. M. Johnson, M. J. Amato, J. Thompson, C. Goodloe, D. Everett, *2017 IEEE Aerospace Conference* (IEEE, 2017), pp. 1–5.

73. M. J. Way, A. D. Del Genio, N. Y. Kiang, L. E. Sohl, D. H. Grinspoon, I. Aleinov, M. Kelley, T. Clune, Was venus the first habitable world of our solar system? *Geophys. Res. Lett.* **43**, 8376–8383 (2016).

74. A. Kramida, Y. Ralchenko, J. Reader, NIST ASD Team, NIST atomic spectra database lines form. *NIST Atomic Spectra Database (ver. 5.2) (Online)* (2014).

75. T. D. Swindle, Martian noble gases. *Rev. Mineral. Geochem.* **47**, 171–190 (2002).

76. D. D. Bogard, D. H. Garrison, Relative abundances of argon, krypton, and xenon in the martian atmosphere as measured in martian meteorites. *Geochim. Cosmochim. Acta* **62**, 1829–1835 (1998).

77. J. Connerney, M. Acuña, N. Ness, G. Kletetschka, D. Mitchell, R. Lin, H. Reme, Tectonic implications of mars crustal magnetism. *Proc. Natl. Acad. Sci. U.S.A.* **102**, 14970–14975 (2005).

78. J. Gilmour, J. Whitby, G. Turner, Disentangling xenon components in nakhla: Martian atmosphere, spallation and martian interior. *Geochim. Cosmochim. Acta* **65**, 343–354 (2001).

79. A. Ciucci, M. Corsi, V. Palleschi, S. Rastelli, A. Salvetti, E. Tognoni, New procedure for quantitative elemental analysis by laser-induced plasma spectroscopy. *Appl. Spectrosc.* **53**, 960–964 (1999).

80. J.-G. Xie, B. Luo, D. Lo, Electron-ion recombination in high pressure Ar/Xe mixtures. *J. Phys. B* **24**, 3077–3089 (1991).

81. A. J. Kelly, Atom—Atom ionization cross sections of the noble gases—Argon, krypton, and xenon. *J. Chem. Phys.* **45**, 1723–1732 (1966).

82. H. Petschek, S. Byron, Approach to equilibrium lonization behind strong shock waves in argon. *Ann. Phys. Rehabil. Med.* **1**, 270–315 (1957).

83. M. I. Hoffert, Precursor ionization effects on magnetohydrodynamic switch-on shock structure. *J. Plasma Phys.* **4**, 477–494 (1970).

84. Y. B. Zel'dovich, Y. P. Raizer, *Physics of Shock Waves and High-Temperature Hydrodynamic Phenomena* (1967).

85. T. Owano, C. Kruger, R. Beddini, Electron-ion three-body recombination coefficient of argon. *AIAA J.* **31**, 75–82 (Elsevier Academic Press; 1993).

86. X. Zeng, X. Mao, R. Greif, R. Russo, Experimental investigation of ablation efficiency and plasma expansion during femtosecond and nanosecond laser ablation of silicon. *Appl. Phys. A* **80**, 237–241 (2005).
